# Chemically Driven Contraction and Elongation: Interconversion between Molecular Figure of Eight

**DOI:** 10.1002/open.202500081

**Published:** 2025-03-20

**Authors:** Sanjaya Kumar Moharana, Radhakrishna Ratha, Chandra Shekhar Purohit

**Affiliations:** ^1^ School of Chemical Sciences National Institute of Science Education and Research (NISER), Jatni 752050 Bhubaneswar Odisha India; ^2^ Homi Bhabha National Institute (HBNI) Mumbai 400 04 Maharashtra India

**Keywords:** Interconversion, figure of eight, Cobalt (III), Copper (I), Macrocycle, De-metalation, Re-metalation

## Abstract

A 90‐membered macrocycle having two functional moiety, diphenyl‐phenanthroline (dpp) and two tri‐dentate pyridine‐diamide (pda) as templating center has been synthesized from precursor molecules via amide bond formation in 10 steps. dpp and pda are arranged alternatively around the macrocycle in a sequence of dpp‐pda‐dpp‐pda and demeostrated orthogonal behaviour, when treated with transition metal such as Cu(I) and Co(III): It forms figure of eight complexes selectively between dpp units and pda units respectively due to the geomtric preferance. This makes the macrocycle operates in bimodal manner. Interconversion between figure of eight complexes has been witnessed by metal‐exchange via demetalation and subsequent re‐metalation between orthogonal templating centres. Removal of Cu(I) template is performed using a common and less toxic reagent NH_2_CH_2_CH_2_NH_2_ and Co(III) by Zn/CH_3_COOH at RT.

## Graphical abstract

A 90‐membered bi‐modal macrocycle functionalized with two dpp and two pda templating centres has been synthesized. When reacted with Co(III) and Cu(I), two figure of eight geometries are formed. Metalation‐demetalation‐remetalation results interconversion between these figure of eight geometries. This process results in orthogonal contraction and elongation of macrocycle and reminiscent of molecular muscle like component.

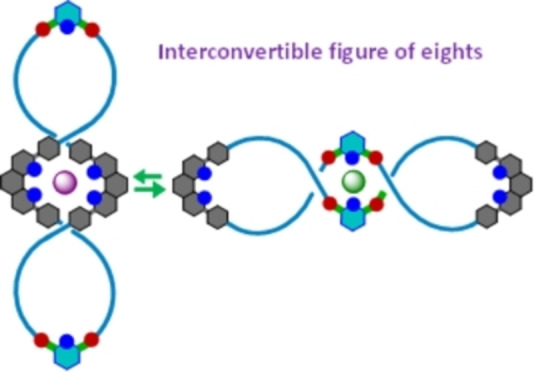

## Introduction

Stimuli responsive motions are vital in living organism to perform biological functions. All these motions originate at molecular level and channelized in to specific modes of molecular movement. One such example is the elongation of muscle fibres in response to various stimuli.^[1]^ This inspires chemists to design and synthesize various molecular systems to replicate such natural functions. The discovery of mechanically interlocked molecules (MIMs), where components can move independently to each other fuelled such endeavour.[^2^, ^3^, ^4^] Examples of artificial/synthetic molecular machines (AMM) includes catenane as molecular switch^[5]^ and motor.[^6^, ^7^, ^8^] Rotaxane acts as molecular pump^[9]^ and linear‐rotaxane dimer shows muscle like behaviour,[^10^, ^11^, ^12^, ^13^] and molecular elevator.^[14]^ These AMMs can be operated by external stimulus to perform mechanical translocation or unidirectional rotation among its interlocked components. Chemical stimuli^[9]^ pH,^[15]^ acid‐base,^[16]^ halide ion,[^17^, ^18^] electrochemical oxidation‐reduction,^[19]^ electric current[^5^, ^20^] and transition metal ion exchange.^[10]^ It may be noted that the functional part is/are carefully tailored in MIMs to become stimuli responsive.[^6^, ^11^, ^19^]

Although several stratgey have been developed for MIMs, transition metals as template is one of the popular methods. Recently complex knots,^[21]^ links,^[22]^ weaving of molecular strands[^23^, ^24^] and molecular fabric has been demonstrated,^[25]^ are made using this strategy. Moreover, metal‐exchange in these MIMs imparts structure‐property relationship such as catalysing chemical reaction,^[26]^ changing mechanical properties of gel^[27]^ and polymer.^[28]^ Further, coordinating centres can be locked in various conformations when metal ion coordinates, leading to controlled translocation of molecular components.[^29^, ^30^] One such example is the linear rotaxane‐dimer from Sauvage's group, having terpyridine and phenanthroline templating centre operated by Zn(II), Cu(I) metal exchange.^[10]^ The shift in coordination environment makes the molecule either shrink or elongate similar to muscle in living organism. Leigh group performed half rotation in a hetero‐circuit [2]catenane having pyridine‐diamide and pyridine templating centre by utilizing Pd(II) and Co(III) metal ion exchange.^[29]^

Apart from MIMs, large macrocycles, which can adopt different shapes, can results in large amplitude response in a controlled manner to external stimuli such as visible light[^31^, ^32^] and metal ion(s).[^33^, ^34^] If reversible, such change in conformation/structure may be compared to component of molecular muscle. The only examples of such behaviour with metal ions as stimuli are Sauvage group's bimodal macrocycles having terpyridine and diphenyl‐phenanthroline as metal binding centres. They have studied interconversion of figure of 8 to ∞ and vice‐versa using Fe(II), Cu(II) and Cu(I) metal exchange as stimuli.[^33^, ^34^]

In our previous works we have demonstrated base‐mediated complexation of Co(III) with pyridine‐diamide templating centre makes it promising candidate for the synthesis of higher order catenane.[^35^, ^36^, ^37^] Having octahedral preference pda can be used in combination with a tetrahedral preferred templating centre such as dpp and serve as a station to construct molecular machine like component. Herein, we report a bimodal macrocycle, under the influence Co(III) and Cu(II), adopting two different figure of eight geometries.^[38]^ Figure of eights are interconvertible by metal exchange. This constitutes orthogonal contraction and elongation between assemblies and reminiscent of molecular muscle like movement.

## Results and Discussion

### (a) Design and Synthesis of Bi‐Modal Macrocycle MC‐b

**Figure 1:**
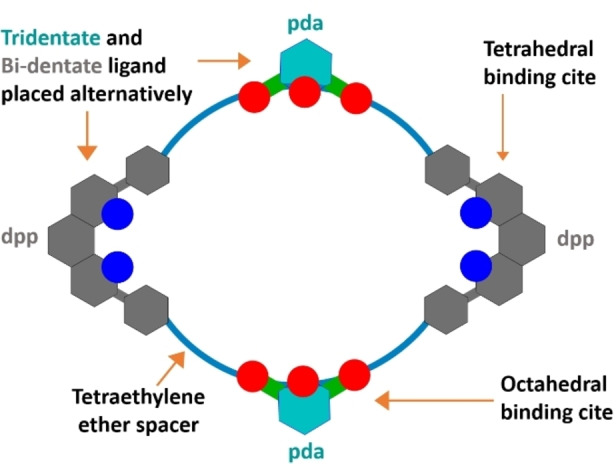
Schematic representation of macrocycle **MC‐b** showing oppositely positioned dpp and pda orthogonal templating centres. Blue circle represent tetrahedral binding site and red circle represent octahedral binding site.

Bi‐modal macrocycle namely **MC‐b** has been designed and synthesized to have two oppositely positioned dpp and two pda templating centres and with four tetraethylene ether chains as spacer (Figure 1). Choice of long tetraethylene ether chains as spacer is to provide solubility in common organic solvents and flexibility.^[31]^ Additionally, it helps the synthetic precursor molecule, in attaining angular geometry for cyclization to occur.^[39]^ Synthesis of **MC‐b** was performed by stepwise joining of diphenyl‐phenanthroline derivative having amine terminal with 2,6‐pyridine‐dicarbonyldichloride through amide bond formation in 10 steps from commercial available compounds (supplementary Scheme SI‐1). All monomers, macrocycle has been characterized by ^1^H‐NMR, ^13^C‐NMR, mass spectroscopy and discussed in supporting information. Phen‐OH (**M2**) has been synthesized following reported literature procedure using Lithium metal at 0 °C.

### (b) ^1^H‐NMR Signal Change during Synthesis of Figure of Eights

**Figure 2:**
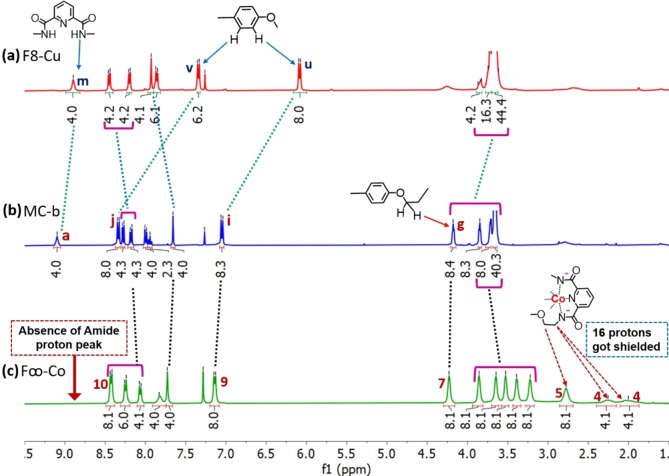
Partial ^1^H‐NMR (400 MHz, CDCl_3_, at 298 K) comparison between macrocycle and figure of eight (a) **F8‐Cu** (b) **MC‐b** (c) **F∞‐Co**. Individual spectral analysis given in supporting information. The assigned numbers and letters are as shown in Scheme 1.

^1^H‐NMR spectrum is recorded in CDCl_3_ for **MC‐b**, **F8‐Cu** and **F∞‐Co**. Four amide protons of **MC‐b** from pda appears at 9.09 ppm (peak **a**, Figure 2b) and peak for benzene protons of dpp units can be seen at 8.34 and 7.06 ppm (peak **j** and **i**, Figure 2b). When treated with Cu(I) and Co(III), distinguished change in proton signal has been observed.

Upon complexation with Cu(I) metal ion, shielding of benzene protons at the vicinity of dpp has been observed, as a result peak **j** shifted to peak **v** (7.35 ppm) and peak **i** shifted to peak **u** (6.09 ppm). Such type of up‐field shifting after Cu(I) complexation has been observed in previously reported literature.[^33^, ^34^] Shielding effect has also been observed for the aliphatic protons next to benzene ring (peak **g**, Figure 2a). In addition, minor de‐shielding and peak shifting has been observed for the phenanthroline ring protons, possible due to twisted geometry^[40]^ of **F8‐Cu**. Amide protons can be observed at 8.9 ppm (peak **m**, Figure 2a), indicates non‐participation of pda during Cu(I) complexation. It is worth to mention that due to paramagnetic nature of Cu(I) complex quenching in proton integration for the benzene proton in close vicinity occurs and shows integration for six protons rather than eight (peak **v**, Figure 2a).

When **MC‐b** reacted with Co(III) metal ion,^[41]^ complexation occurs at pda and hence four amide protons are now absent in **F∞‐Co** (Figure 2c). Peaks for the dpp unit remains unchanged in **F∞‐Co** (peak **9**, **10**, Figure 2c), suggest non‐participation of dpp unit. Moreover, 16‐aliphatic protons in close vicinity of Co(III) complex (−N^−^−**CH_2_**−**CH_2_**−O−) got shielded (peak **4**, **5**, Figure 2c) due to perturbation in the geometry. Similar shielding effect has also been observed in our previously synthesized Co(III) catenates.[^35^, ^36^] Other protons of tetraethylene ether chain in **F∞‐Co** also suffer minor up‐field shift and appears in between 3.86 to 2.78 ppm (Figure 2c) compared to **MC‐b** (Figure 2b). No change is seen for the aliphatic proton next to benzene (peaks **g** shifted to peak **7**, Figure 2b, **2 c** respectively) due to non‐participation of the dpp unit.

### (c) ^13^C‐NMR signal Change during Synthesis of Figure of Eights

**Figure 3:**
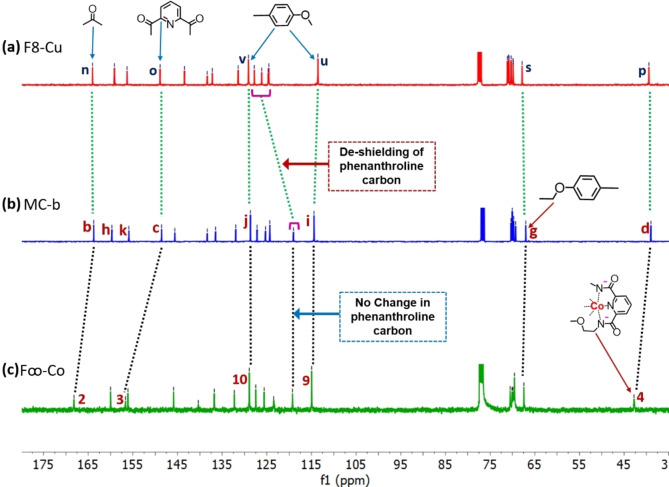
Partial ^13^C‐NMR (400 MHz, CDCl_3_, at 298 K) comparison between macrocycle and figure of eights (a) **F8‐Cu** (b) **MC‐b** (c) **F∞‐Co**. Individual spectral analysis given in supporting information. The assigned numbers and letters can are as shown in Scheme 1.

Formation of **F8‐Cu** and **F∞‐Co** can also be evidenced from ^13^C‐NMR spectra. In CDCl_3_ **MC‐b** shows peaks for carbonyl carbon of pda unit at 163.93 ppm (peak **b**, Figure 3b) and benzene proton attached to dpp unit at 127.42 ppm (peak **j**, Figure 3b) and 114.69 ppm (peak **i**, Figure 3b). Upon complexation with Cu(I), minor peak shift is seen for the benzene protons at close vicinity: peaks **j** and **i** of Figure 3b are seen as peak **v** and **u** respectively in Figure 3a. However, de‐shielding of dpp ring carbon at 119.29 ppm **(**Figure 3b **)** was observed due to complexation. No change for the aliphatic carbons was observed. Moreover, no change in the carbonyl carbon peak suggests non‐participation of pda unit in Cu(I) complexation. When **MC‐b** is complexed with Co(III),^[41]^ de‐shielding of carbonyl carbon (peak **b**, Figure 3b) and pyridine carbon attached to carbonyl group (peak **c**, Figure 3b) was observed. This type of de‐shielding caused due to complexation was also observed in our previously synthesized Co(III) catenantes.[^35^, ^36^] Moreover, aliphatic carbon next to Co(III) complex also suffers perturbation and can be observed in ^13^C‐NMR (peak **4**, Figure 3c). No change in dpp carbon signal (Figure 3c) and benzene carbon signal in **F∞‐Co** (Figure 3c) suggest Co(III) Co(III) is near the pda units only.

### (d) Mass Spectral Change during Synthesis of F8‐Cu and F∞‐Cu

Positive mode ESI‐mass spectra of **MC‐b** (C_94_H_102_ N_10_O_20_) shows one prominent peak with isotopic distribution pattern at m/z 846.885 and corresponds to a di‐cationic species. This peak is attributed to [**MC‐b**+2H^+^]^2+^ with calculated m/z for [C_94_H_104_N_10_O_20_]^2+^ is 846.8712. The molecular ion peak for [**MC‐b**+H^+^]^+^ can also be observed at m/z 1692.6823 and matching with the calculated m/z for (C_94_H_103_ N_10_O_20_)^+^: 1692.7349. The complex obtained from **MC‐b** with Cu(I) metal results in a prominent di‐cationic peak at 888.8824. This matches with the calculated m/z for [**F8‐Cu**+Na]^2+^ [C_94_H_102_CuN_10_O_20_Na]^2+^: 888.8230. Also a mono cationic peak is seen at m/z 1755.946, and assigned to [**F8‐Cu**+H]^+^ that matches with the simulated m/z for (C_94_H_103_CuN_10_O_20_)^+^] 1755.667. Likewise, when **MC‐b** complexed with Co(III) metal ion, a di‐cationic peak at 885.8425 was observed which is attributed to [**F∞‐Co**+2H+Na]^2+^. This is in good agreement with corresponding calculated m/z for (C_94_H_100_CoN_10_O_20_Na)^+^ 885.819 (supplementary Figure S29–S31).

### (e) Interconversion between F8 and F∞ using Chemical Stimuli

**Scheme 1:**
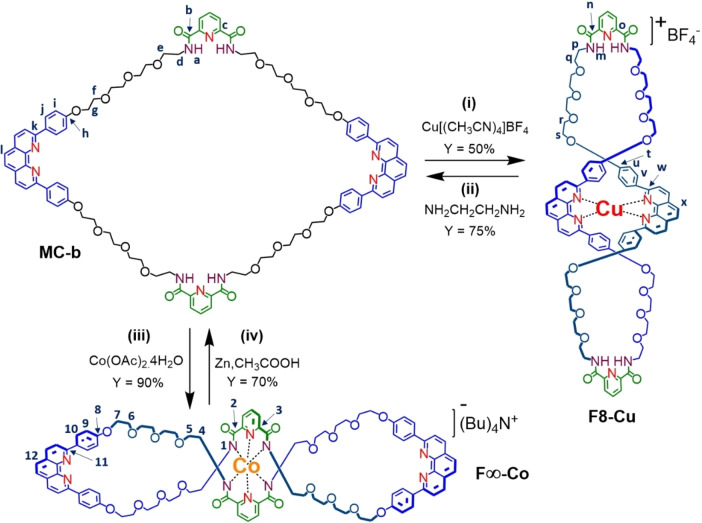
Reaction scheme for synthesis of **F8‐Cu** and **F∞‐Co** form bi‐modal macrocycle **MC‐b** and Interconversion between them (i) Cu(CH_3_CN)_4_,BF_4_, DCM:CH_3_CN(1 : 1), RT, 2 h (ii) NH_2_CH_2_CH_2_NH_2_, DCM, RT, 5 min. (iii) Co(OAc)_2_.4H_2_O, NaH, MeOH, reflux, 12 h (iv) Zn dust, AcOH:MeOH (1 : 1), RT, 5 h. Synthetic procedures and detailed characterizations of **MC‐b** and metal complexes are given in supporting information. For simplification, at one time, figure of eight from copper complex is represented a vertical isomer **F8‐Cu**, hence the cobalt complex is represented as corresponding horizontal isomer **F∞‐Co**.

Interconversion between **F8‐Cu** and **F∞‐Co** is carried out by metal exchange. When **MC‐b** is treated with [Cu(CH_3_CN)_4_]BF_4_^−^ in DCM : CH_3_CN at RT, reaction takes place between two bi‐dentate dpp units only, forming a stable red‐black coloured figure of eight Cu‐complex **F8‐Cu** in 50 % yield and having tetrahedral geometry (Scheme 1 **)**. The low yield is due to other unknown Cu(I) complex formation. Demetalation of Cu(I) from **F8‐Cu** complex was performed using ethylene diamine (NH_2_CH_2_CH_2_NH_2_) in DCM at RT for 5 minutes results in reversal of **F8‐Cu** to its synthetic precursor macrocycle **MC‐b** in 75 % yield.^[42]^ Immediate disappearance of red‐black colour suggest removal of Cu(I). Isolated **MC‐b** from demetalation of **F8‐Cu** is further reacted with NaH and Co(OAc)_4_.4H_2_O in MeOH.^[36]^ This time, complexation occurs between two pda units only, forming a yellow‐green figure of eight Co‐complex **F8‐Co** having octahedral geometry in 90 % yield. Demetalation of **F8‐Co** using Zn/CH_3_COOH at RT for 5 hours results disappearance of yellow‐green colour and formation of starting material **MC‐b** in 70 % yield. Demetalation **F8‐Cu**, lead to formation of macrocycle **MC‐b** and upon re‐complexation using orthogonal metal ion Co(III) leads to switching of vertically oriented **F8‐Cu** to its horizontal isomer **F∞‐Co** and vice‐versa.^[33]^ During metalation and de‐metalation the macrocycle undergoes elongation, relaxation and contraction.[^33^, ^34^] This reminiscent of molecular muscle like assembly.

### (f) Optical Properties of F8‐Cu, MC‐b and F∞‐Co

**Figure 4:**
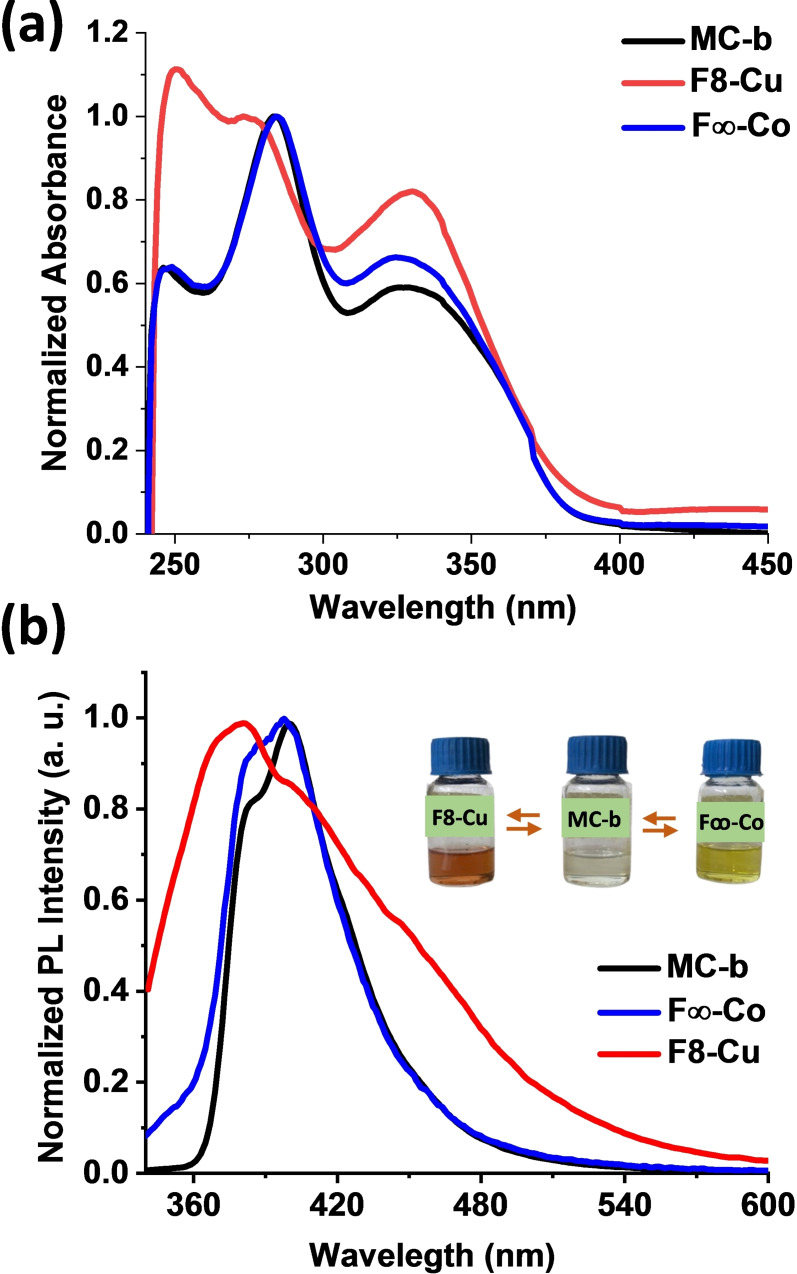
(a) UV‐visible spectra (b) fluorescence spectra with photographic images of chloroform solution of **MC‐b**, **F8‐Cu** and **F∞‐Co**. Interconversion from **F8‐Cu** and **F∞‐Co** or vice versa is also witnessed by change in colour of the solution.

Absorbance spectra were recorded from anhydrous CHCl_3_ solution (Figure 4a). Both **MC‐b** and **F∞‐Co** demonstrates two λ_max_ at ≈330 nm and ≈282 nm. For **F8‐Cu**, lower wavelength maximum was blue shifted by 10 nm and appears at 274 nm. Such blue shift in Cu(I) phenanthroline complex can be attributed to change in π‐π stacking interaction between dpp units of **F8‐Cu**.^[43]^ Corresponding fluorescence spectra were recorded by exciting at 282 nm. Both figure of eight geometries demonstrated distinguished emission maximum. **F8‐Cu** shows λ_em_ at 382 nm, whereas for **F∞‐Co** λ_em_ appears at 400 nm. Minor spectral change is observed when **MC‐b** is complexed at pda forming **F∞‐Co**. However when dpp unit are complexed to form **F8‐Cu**, a blue shift of 18 nm is observed with broad spectra (Figure 4b).

Blue shift can be attributed to the extended conjugation unit present in dpp unit compared to pda and change in π–π stacking^[44]^ interactions between dpp units of **F8‐Cu** in solution. Also, spectral broadening observed for Cu(I) complexed **F8‐Cu** can be attributed to formation of lower energy MLCT transitions. As a result, both the figure of eight complexes has distinguished color compared to bi‐modal macrocycle F8‐Cu is red‐black in color whereas, F∞‐Co is greenish‐yellow in colour. Hence the interconversion between figure of eights can be monitored in naked eye during reaction.

## Conclusions

Interconversion between two molecular figure of eight geometry has been demonstrated via a bi‐modal macrocycle by using transition metal Cu(I) and Co(III) metalation‐demetalation‐remetalation. Interconversion can be observed in naked eye during the experiment, due to colour change from deep‐red Cu(I) complex to green coloured Co(III) complex or vice‐versa. Moreover, **F8‐Cu** and **F∞‐Co** shows distinguished ^1^H‐NMR spectra in CDCl_3_ due to presence and absence of amide bond respectively. Synthesized figure of eight has different charge state: **F8‐Cu** is +1 charged and **F∞‐Co** is −1 charged. Hence can be distinguished by –ve mode mass spectrometry. Such bi‐modal macrocycle design can be used to design molecular muscle like machine. This study further establish pda and dpp as orthogonal templating centre that can be used to build stations in other AMM and MIMs.

## Conflict of Interests

The authors declare no conflict of interest.

## Supporting information

As a service to our authors and readers, this journal provides supporting information supplied by the authors. Such materials are peer reviewed and may be re‐organized for online delivery, but are not copy‐edited or typeset. Technical support issues arising from supporting information (other than missing files) should be addressed to the authors.

Supporting Information

## Data Availability

The data that support the findings of this study are available in the supplementary material of this article.

## References

[1] F. Lancia , A. Ryabchun , N. Katsonis , Nat. Rev. Chem. 2019, 3, 536–551. 10.1038/s41570-022-00392-8 37117430

[2] Z. Meng , Y. Han , L. N. Wang , J. F. Xiang , S. G. He , C. F. Chen , J. Am. Chem. Soc. 2015, 137, 9739–9745. 26186017 10.1021/jacs.5b05758

[3] M. Gauthier , K. F. Marotte , C. Clavel , P. Waeles , P. Laurent , F. Coutrot , Angew. Chem. 2023, 62, e202310643. 37594476 10.1002/anie.202310643

[4] S. E. Cakmak , D. A. Leigh , C. T. M. Ternan , A. L. Nussbaumer , Chem Rev. 2015, 115, 10081–10206. 26346838 10.1021/acs.chemrev.5b00146 PMC4585175

[5] C. P. Collier , G. Mattersteig , E. W. Wong , Y. Luo , K. Beverly , J. Sampaio , F. M. Raymo , J. F. Stoddart , J. R. Heath , Science 2000, 289, 1172–1175. 10947980 10.1126/science.289.5482.1172

[6] L. Zhang , Y. Qiu , W. G. Liu , H. Chen , D. Shen , B. Song , K. Cai , H. Wu , Y. Jiao , Y. Feng , J. S. W. Seale , C. Pezzato , J. Tian , Y. Tan , X. Y. Chen , Q. H. Guo , C. L. Stern , D. Philp , R. D. Astumian , W. A. Goddard , J. F. Stoddart , Nature 2023, 613, 280–286. 36631649 10.1038/s41586-022-05421-6 PMC9834048

[7] M. R. Wilson , J. Solà , A. Carlone , S. M. Goldup , N. Lebrasseur , D. A. Leigh , Nature 2016, 534, 235–240. 27279219 10.1038/nature18013

[8] S. Erbas-Cakmak , S. D. P. Fielden , U. Karaca , D. A. Leigh , C. T. McTernan , D. J. Tetlow , M. R. Wilson , Science 2017, 358, 340–343. 29051374 10.1126/science.aao1377

[9] J. Berná , D. A. Leigh , M. Lubomska , S. M. Mendoza , E. M. Pérez , P. Rudolf , G. Teobaldi , F. Zerbetto , Nat. Mate. 2005, 4, 704–710. 10.1038/nmat1455 16127455

[10] M. C. Jiménez , C. D. Buchecker , J. P. Sauvage , Angew. Chem. 2000, 39, 3284–3287. 11028078 10.1002/1521-3773(20000915)39:18<3284::aid-anie3284>3.0.co;2-7

[11] Y. Liu , A. H. Flood , P. A. Bonvallet , S. A. Vignon , B. H. Northrop , H. R. Tseng , J. O. Jeppesen , T. J. Huang , B. Brough , M. Baller , S. Magonov , S. D. Solares , W. A. Goddard , C. M. Ho , J. F. Stoddart , J. Am. Chem. Soc. 2005, 127, 9745–9759. 15998079 10.1021/ja051088p

[12] S. J. Rao , X. H. Ye , Q. Zhang , C. Gao , W. Z. Wang , D. H. Qu , Asian J. Org. Chem. 2018, 7, 902–905.

[13] Q. Zhang , S. J. Rao , T. Xie , X. Li , T. Y. Xu , D. W. Li , D. H. Qu , Y. T. Long , H. Tian , Chem 2018, 4, 2670–2684.

[14] J. D. Badjić , V. Balzani , A. Credi , S. Silvi , J. F. Stoddart , Science 2004, 303, 1845–1849. 15031499 10.1126/science.1094791

[15] F. Coutrot , C. Romuald , E. Busseron , Org. Lett. 2008, 10, 3741–3744. 18666774 10.1021/ol801390h

[16] G. Du , E. Moulin , N. Jouault , E. Buhler , N. Giuseppone , Angew. Chem. 2012, 51, 12504–12508. 23081866 10.1002/anie.201206571

[17] C. J. Chuang , W. S. Li , C. C. Lai , Y. H. Liu , S. M. Peng , I. Chao , S. H. Chiu , Org. Lett. 2009, 11, 385–388. 19099497 10.1021/ol802648h

[18] M. Jaiswal , S. Dasgupta , Org. Lett. 2024, 26, 6776–6781. 39053506 10.1021/acs.orglett.4c02544

[19] J. P. Collin , F. Durola , J. Lux , J. P. Sauvage , Angew. Chem. 2009, 48, 8532–8535. 19798707 10.1002/anie.200903311

[20] J. E. Green , J. W. Choi , A. Boukai , Y. Bunimovich , E. J. Halperin , E. DeIonno , Y. Luo , B. A. Sheriff , K. Xu , Y. Shik Shin , H. R. Tseng , J. F. Stoddart , J. R. Heath , Nature 2007, 445, 414–417. 17251976 10.1038/nature05462

[21] J. Zhong , Z. Sun , L. Zhang , G. F. S. Whitehead , I. J. V. Yrezabal , D. A. Leigh , J. Am. Chem. Soc. 2024, 146, 21762–21768. 39060953 10.1021/jacs.4c05953 PMC11311214

[22] N. D. Colley , M. A. Nosiglia , S. L. Tran , G. H. Harlan , C. Chang , R. Li , A. O. Delawder , Y. Zhang , J. C. Barnes , ACS Cent. Sci. 2022, 8, 1672–1682. 36589894 10.1021/acscentsci.2c00697 PMC9801505

[23] Y. Liu , Y. Ma , Y. Zhao , X. Sun , F. Gándara , H. Furukawa , Z. Liu , H. Zhu , C. Zhu , K. Suenaga , P. Oleynikov , A. S. Alshammari , X. Zhang , O. Terasaki , O. M. Yaghi , Science 2016, 351, 365–369. 26798010 10.1126/science.aad4011

[24] G. Li , J. Zhao , Z. Zhang , X. Zhao , L. Cheng , Y. Liu , Z. Guo , W. Yu , X. Yan , Angew. Chem. 2022, 61, e202210078. 36047492 10.1002/anie.202210078

[25] D. P. August , R. A. W. Dryfe , S. J. Haigh , P. R. C. Kent , D. A. Leigh , J. F. Lemonnier , Z. Li , C. A. Muryn , L. I. Palmer , Y. Song , G. F. S. Whitehead , R. J. Young , Nature 2020, 588, 429–435. 33328664 10.1038/s41586-020-3019-9

[26] A. Bessaguet , Q. B. Remaury , P. Poinot , I. Opalinski , S. Papot , Angew. Chem. 2023, 62, e202216787. 36478644 10.1002/anie.202216787 PMC10107136

[27] M. A. Nosiglia , N. D. Colley , M. K. Danielson , M. S. Palmquist , A. O. Delawder , S. L. Tran , G. H. Harlan , J. C. Barnes , J. Am. Chem. Soc. 2022, 144, 9990–9996. 35617307 10.1021/jacs.2c03166

[28] R. Bai , Z. Zhang , W. Di , X. Yang , J. Zhao , H. Ouyang , G. Liu , X. Zhang , L. Cheng , Y. Cao , W. Yu , X. Yan , J. Am. Chem. Soc. 2023, 145, 9011–9020. 37052468 10.1021/jacs.3c00221

[29] D. A. Leigh , P. J. Lusby , A. M. Z. Slawin , D. B. Walker , Chem. Commun. 2012, 48, 5826–5828. 10.1039/c2cc32418k 22572809

[30] D. A. Leigh , P. J. Lusby , A. M. Z. Slawin , D. B. Walker , Chem. Commun. 2005, 4919–4921, DOI: 10.1039/B510663J. 16205799

[31] Y. Liu , Q. Zhang , S. Crespi , S. Chen , X. K. Zhang , T. Y. Xu , C. S. Ma , S. W. Zhou , Z. T. Shi , H. Tian , B. L. Feringa , D. H. Qu , Angew. Chem. 2021, 60, 16129–16138. 33955650 10.1002/anie.202104285 PMC8361693

[32] E. Nieland , J. Voss , A. Mix , B. M. Schmidt , Angew. Chem. 2022, 61, e202212745. 36165240 10.1002/anie.202212745 PMC9828355

[33] F. Niess , V. Duplan , J. P. Sauvage , J. Am. Chem. Soc. 2014, 136, 5876–5879. 24712650 10.1021/ja501765y

[34] F. Niess , V. Duplan , C. S. Diercks , J. P. Sauvage , Chem. Eur. J. 2015, 21, 14393–14400. 26332007 10.1002/chem.201502216

[35] M. B. Podh , R. Ratha , C. S. Purohit , Chem. Asian J. 2024, 19, e202400031. 38372572 10.1002/asia.202400031

[36] M. B. Podh , R. Ratha , C. S. Purohit , Chem. Asian J. 2024, 19, e202400351. 38700467 10.1002/asia.202400351

[37] P. Ghosh , R. Ratha , C. S. Purohit , Chem. Asian J. 2024, 19, e202400668. 39082610 10.1002/asia.202400668

[38] S. Prusty , M. B. Podh , C. S. Purohit , ChemistrySelect 2018, 3, 9690–9693.

[39] J. H. May , J. M. Fehr , J. C. Lorenz , L. N. Zakharov , R. Jasti , Angew. Chem. 2024, 63, e202401823. 38386798 10.1002/anie.202401823

[40] K. Matsumura , K. Kinjo , K. Tateno , K. Ono , Y. Tsuchido , H. Kawai , J. Am. Chem. Soc. 2024, 146, 21078–21088. 39029122 10.1021/jacs.4c06560 PMC11295176

[41] Reaction occurs with Co(II) metal ion, which later oxidized to Co(III) upon exposure to air.

[42] Y. Yao , Y. Deng , L. Kong , H. Y. A. Yeung , Eur. J. Inorg. Chem. 2022, e202200271.

[43] J. L. Appleton , L. Ballerini , S. Choua , C. Gourlaouen , R. Ruppert , M. Mauro , Eur. J. Inorg. Chem. 2024, 27, e202400278.

[44] M. M. Montoya , R. A. J. Janssen , Adv. Funct. Mater. 2017, 27, 1605779.

