# Supplementary material for: Chemically Driven Contraction and Elongation: Interconversion between Molecular Figure of Eight
Source: ChemistryOpen. 2025 Mar 20;14(9):e202500081. doi: 10.1002/open.202500081 (PMC12409827; doi:10.1002/open.202500081)
Supplement: Supplementary file 1 — Supporting Information [file OPEN-14-e202500081-s001.pdf]

ChemistryOpen

Supporting Information

Chemically Driven Contraction and Elongation: Interconversion between Molecular Figure of Eight

Sanjaya Kumar Moharana, Radhakrishna Ratha, and Chandra Shekhar Purohit*

Supporting Information

Chemically Driven Contraction and Elongation: Interconversion between Molecular Figure of Eights

Sanjaya Kumar Moharana,[#, a,b] Radhakrishna Ratha,[#, a,b] Chandra Shekhar Purohit*[, a,b]

# Authors Contributed Equally

[a] *School of Chemical Sciences, National Institute of Science Education and Research (NISER), Jatni, 752050, Bhubaneswar, Odisha, India. E-mail: purohit@niser.ac.in*

[b] *Homi Bhabha National Institute (HBNI), Mumbai, 400 04, Maharashtra, India*

Content

Section-1 Reagents and Instruments

Section-2 Reaction Scheme

Section-4 Synthetic Procedure

Section-5 Spectral Analysis (^{1}H-NMR, ^{13}C-NMR and Mass spectra)

(a) Reagents and Instruments: $SOCl_2$, Et_3N, ethylenediamine, 18-crown-6, pyridine hydrochloride, P-bromoanisole, 1,10-phenanthroline, Li-metal, $Co(OAc)_2.4H_2O$, anhydrous toluene and NaH has been purchased from Spectrochem India Pvt. Ltd. 2,6-Pyridinedicarboxylic acid, MnO_2 is used from Himedia, India Pvt. Ltd. K_2CO_3, p-tolunesulfonyl choride, NaN_3, 4-dimethylaminopyridine, PPh_3, tetraethylene glycol brought from Sigma Aldrich. CH_3COOH taken from SRL Chemicals and tetrabutylammonium acetate is from Alfa Aeser. $Cu(CH_3CN)_4BF_4$ purchased from Acros Organics. Common organic solvents such as hexane, toluene, Et_2O, EtOAc, CH_3CN, DCM, DMF, THF, MeOH and EtOH purchased from Merck and used without further purification. Silica gel (100-200 mesh) has been used for column chromatography. Anhydrous MeOH, DCM, CH_3CN, THF, DMF has been prepared by common laboratory methods. $CDCl_3$ has been used from Sigma Aldrich. ^{1}H-NMR and ^{13}C-NMR spectra was recorded in a Bruker 400 MHz spectrometer. Mass spectra has been recorded in Water ESI-MS and Bruker-ultraflextreme MALDI-TOF spectrometer. UV-visible spectra were recorded in Jasco spectrophotometer and fluorescence spectra were recorded in agilent cary eclipse fluorescence spectrometer

(b) Reaction Scheme

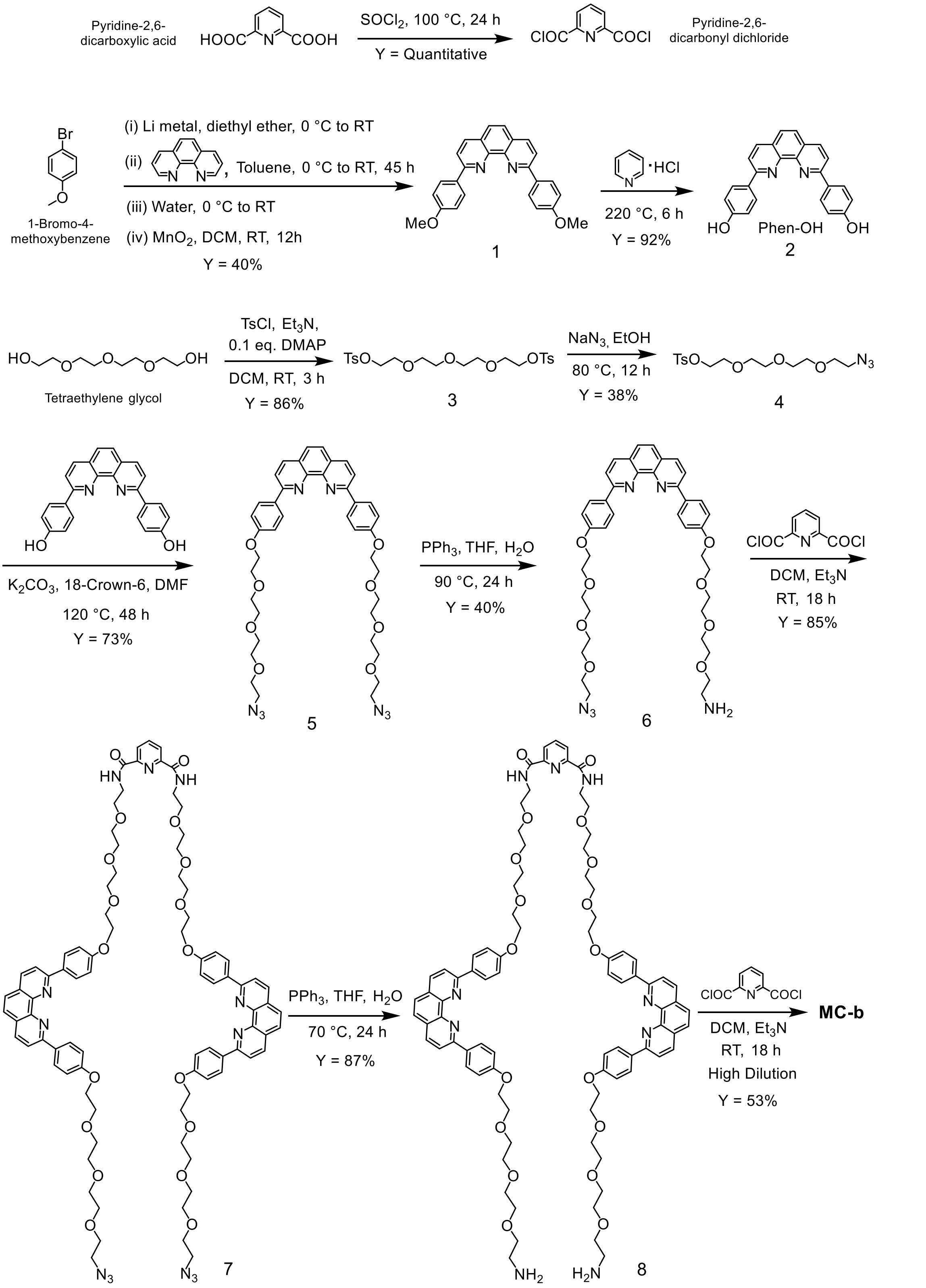

Scheme SI-1. Synthetic scheme for bi-modal macrocycle **MC-b** from commercial available starting material tetraethylene glycol, 2,2'-pyridine-dicarboxylic acid and p-bromoanisole in 10 steps.

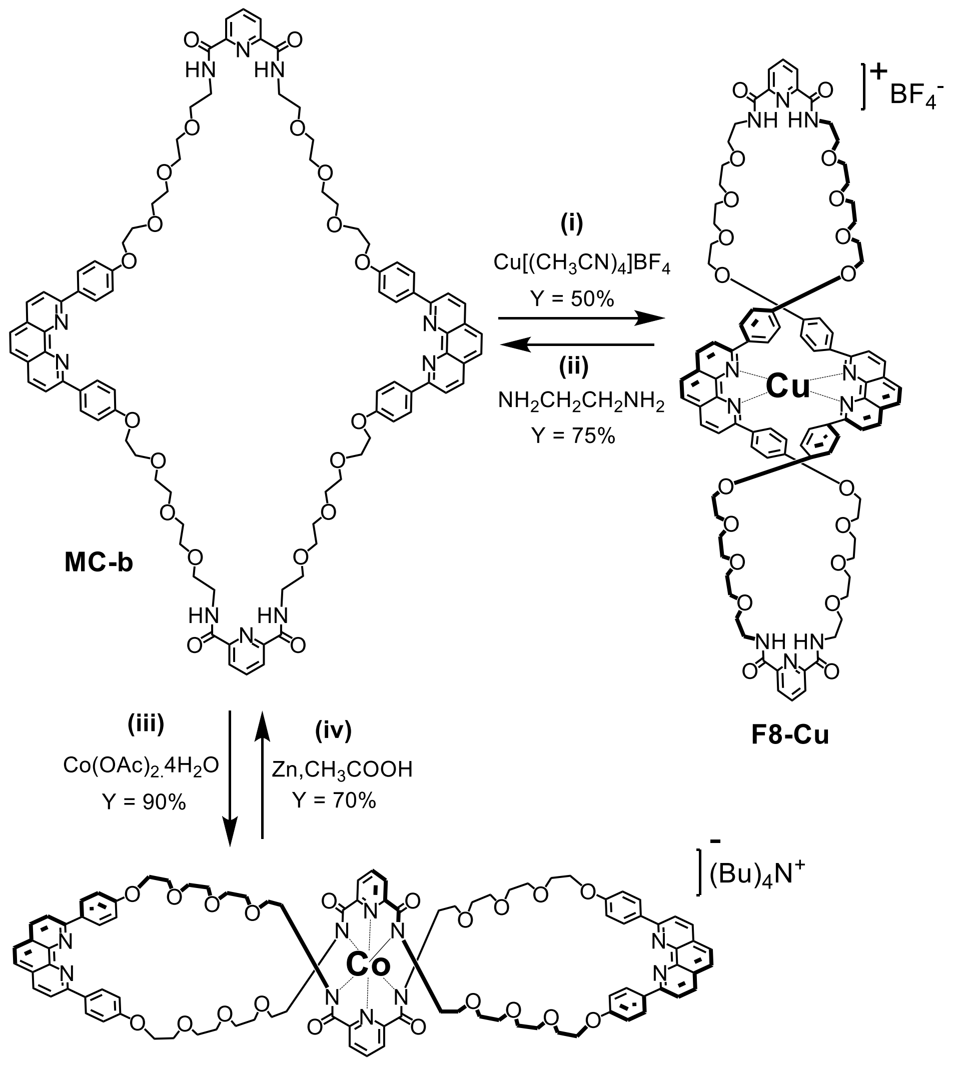

Scheme SI-2. Reaction scheme for synthesis of **F8-Cu** and **F∞-Co** form bi-modal macrocycle **MC-b** and Interconversion between them (i) Cu(CH_3CN)$_4$,BF_4, DCM:CH_3CN(1:1), RT, 2h (ii) $NH_2CH_2CH_2NH_2$, DCM, RT, 5 min. (iii) Co(OAc)$_2$.4H_2O, NaH, MeOH, reflux, 12 h (iv) Zn dust, AcOH:MeOH(1:1), RT, 5h. For simplification, at one time, figure of eight from copper complex is represented a vertical isomer **F8-Cu**, hence the Cobalt complex is represented as corresponding horizontal isomer **F∞-Co**.

(c) Synthetic Procedure:

(1) 2,6-Pyridinedicarbonyl dichloride:

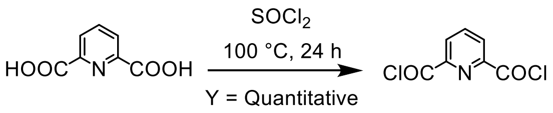

2,6-Pyridinedicarboxylic acid (5 g, 29.9186 mmol) was dissolved in 32 mL of $SOCl_2$ in a 100 mL round bottom flask. This reaction mixture was stirred and refluxed at 120 °C for 24 hours.

Appearance of pink color solution indicates completion of reaction. After cooling to RT, excess $SOCl_2$ was evaporated under high vacuum to yield the desired product 2,6-pyridinedicarbonyl dichloride (white to pink solid) in quantitative yield. The product is moisture and air sensitive and stored in a sealed and argon environment at 4 °C.

(2) Monomer M1:

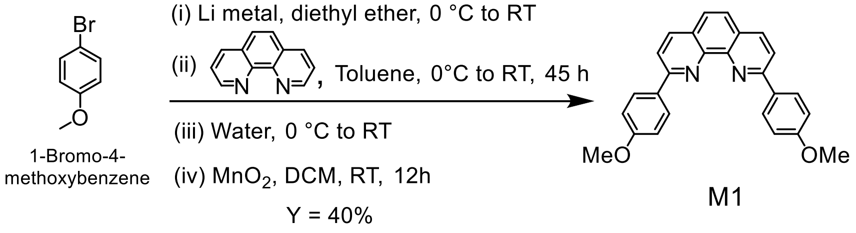

Monomer **M1** was synthesized by following reported literature procedure,[1-3] from p-bromoanisole and 1,10-phenanthroline. A three-neck 250 ml RB flask was taken, fitted with a 100 ml dropping funnel. 3.69 g of freshly and finely cut lithium metal was transferred into it under argon atmosphere. To it, 120 ml of anhydrous diethyl ether was transferred. Then, it was brought to 0 ˚C using ice bath and stirred well to form a suspension. In another 100 ml RB flask, 30 g of p-bromoanisole and 80 ml of anhydrous diethyl ether was transferred under argon atmosphere. This solution was poured into the dropping funnel fitted with the three-neck RB flask under argon atmosphere. Maintaining the temperature at 0 ˚C, 10 ml of p-bromoanisole solution was added at once and stirred for 10 minutes. At this stage shining surface of lithium can be seen, indicates intitiation of reaction. Now, rest of p-bromoanisole solution was added dropwise (1 drop per second) to the suspension of lithium metal in diethyl ether for 1 hour while maintaining the temperature at 0 ˚C. Then, it was slowly brought to RT and stirred for overnight. At this stage p-bromoanisole is converted to 4-methoxyphenyl lithium. In a another 500 ml RB flask, 5 g of anhydrous 1,10-phennanthroline was taken and kept at 125 ˚C inside oven for 30 minutes until it melts and all water gets removed. It was degassed and 200 ml of anhydrous toluene was transferred under argon atmosphere and sonicated to solubilize. This solution was brought to 0 ˚C using ice bath. To it, suspension of 4-methoxyphenyl lithium in diethyl ether was transfer using syringe under argon atmosphere. Initially, it was stirred for 1 h at 0 ˚C and slowly brought to RT and stirred for another 45 hours. This reaction mixture was quenched by slowly adding ice cold water into it while maintaining the outside temperature at 0 ˚C using ice bath. It was brought to RT and stirred for another 1

hour. Further, a work up was performed adding DCM into it. The organic layer was concentrated and 100 g of MnO_2 and 600 ml of fresh DCM was added to it and stirred for 12 hour at RT to re-aromatize. After this, anhydrous Na_2So_4 was added to it and filtered using filter paper at once. The filtrate was concentrated and purified by using silica column chromatography starting from 1:1 hexane: DCM up to 0.5:95.5 MeOH: DCM via 100% DCM. The collected product was washed twice with diethyl ether to get pure product **M1** as pale yellow solid (4.4 g) with yield of 40.41%. **^{1}H-NMR** (400 MHz, $CDCl_3$) δ in ppm: 8.45-8.43 (d, 4H), 8.26-8.24 (d, 2H), 8.09-8.07 (d, 2H), 7.73 (s, 2H), 7.13-7.11 (d, 4H), 3.92 (s, 6H). **^{13}C-NMR** (100 MHz, $CDCl_3$) δ in ppm: 160.86, 156.33, 145.97, 136.76, 132.12, 128.97, 127.50, 125.58, 119.33, 114.16, 55.36.

(3) Monomer M2 (Phen-OH):

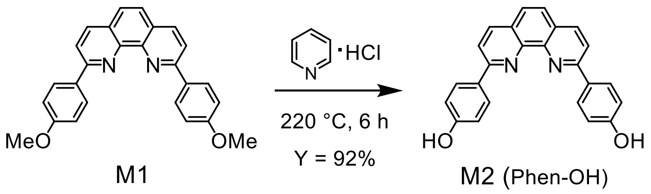

Monomer **M2** was synthesized following literature procedure.[3] To a 500 ml RB flask, 1.5 g (1 equiv., 3.82 mmol) monomer **M1** and pyridine hydrochloride 28.69 g (65 equiv., 248.43 mmol) was transferred**.** It was stirred and refluxed at 220 ˚C for 6 hours under argon atmosphere. Then, it was brought to 180 ˚C and 60 mL of hot water was added into it slowly. Effervescence will appear. Heating process was stopped to bring the reaction mixture to RT. To it, mixed solvent of EtOH and water in 40:60 ratio (50:75 mL) was added. It was stirred, for 1 hour at RT and left at 4 ˚C for overnight. This suspension was neutralized to pH 7.4 exactly, by adding 225 ml of 1M NaOH (prepared by taking 9 g NaOH and making volume up to 225 mL by adding DI water). A change is color from light red to orange was observed. The quenched suspension was left again at 4 ˚C for 2 hours and subsequently filtered to collect the precipitate. the precipitate was washed with a portion of mixed solvent of EtOH:H_2O (40:60, v/v) and dried using tissue paper at RT to get desired product **M2** brown colored solid in 92% yield. **^{1}H-NMR** (400 MHz, DMSO-D_6) δ in ppm: 9.99 (br, 2H), 8.53-8.51 (d, 2H), 8.37-8.35 (d, 4H), 8.30-8.28 (d, 2H), 7.93 (s, 2H), 7.03-7.00 (d, 4H). **^{13}C-NMR** (100 MHz, DMSO-D_6) δ in ppm: 159.38, 155.30, 137.64, 129.07, 127.25, 125.64, 119.55, 115.81.

(4) Monomer M3:

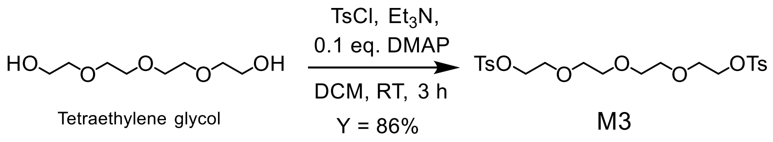

Tetraethylene glycol (20 g, 102.9 mmol), tosyl chloride (78.51 g, 411.8 mmol), triethylamine (172.4 mL, 1235.4 mmol) was dissolved in DCM under argon atmosphere at 0 °C and this reaction mixture was brought to RT and stirred for one hour. Again, it was brought to 0 °C using ice bath and DMAP (1.257 g, 0.1 equivalent, 10.29 mmol) was added into the mixture. This reaction mixture was stirred at RT for 2 hours. Immediately quenched by adding water at 0 °C and extracted with DCM. The combined DCM layer were dried over anhydrous Na_2SO_4, concentrated and purified by column chromatography (with hexane: EtOAc, 50:50 v/v as eluent) to produce pale yellow liquid product **M3** (34 g, 65.7%). **^{1}H-NMR** (400 MHz, $CDCl_3$) δ in ppm: 7.79-7.77 (d, 4H), 7.34-7.32 (d, 4H), 4.16-4.13 (m, 4H), 3.68-3.67 (dd, 4H), 3.55 (s, 8H), 2.43 (s, 6H). **^{13}C-NMR** (100 MHz, $CDCl_3$) δ in ppm: 144.79, 132.94, 129.80, 127.93, 70.68, 70.50, 69.22, 68.64, 21.60. **HR-MS (ESI$^+$):** Calcd. m/z for $C_{22}H_{30}O_9S_2Na$ $[M+Na]^+$ 525.1398, found 525.1376.

(5) Monomer M4:

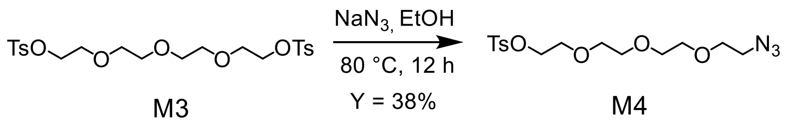

Monomer **M4** was synthesized according to reported literature procedure.[4] Monomer **M3** (4 g, 7.96 mmol) and NaN_3 (0.556 g, 8.56 mmol) were mixed in a 250 mL RB flask and 150 ml absolute ethanol was added into it. The reaction mixture was heated at 80 °C for not more than 12 hours. EtOH was evaporate to dryness under reduced pressure followed by column chromatography using 75:25 hexane: EtOAc as eluent to get desired product (colorless liquid) **M4** in 38% yield (1.13 g). The product formed can be visualize over thin layer chromatography using UV-lamp. **^{1}H-NMR** (400 MHz, $CDCl_3$) δ in ppm: 7.79-7.77 (d, 2H), 7.34-7.32 (d, 2H), 4.14 (t, 2H), 3.68-3.58 (m, 12H), 3.36 (t, 2H), 2.43 (s, 3H). **^{13}C-NMR** (100 MHz, $CDCl_3$) δ in ppm: 144.75, 132.90, 129.75, 127.89, 70.66, 70.56, 70.50, 69.94, 69.18, 68.58, 50.59, 21.55. **HR-MS (ESI$^+$):** Calcd. m/z for $C_{15}H_{23}N_3O_7SNa$ $[M+Na]^+$ 412.1149, found 412.0855.

(6) Monomer M5:

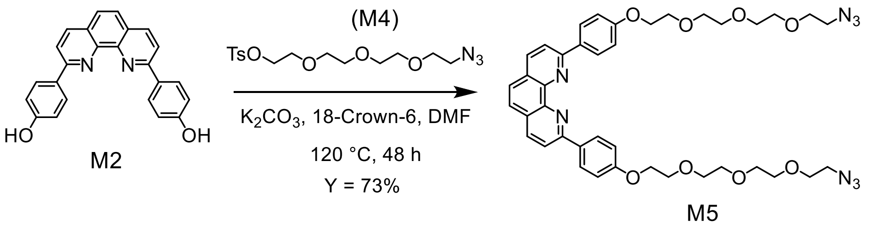

Monomer **M4** (5 g, 13.389 mmol), **M2** (2.244 g, 6.159 mmol), anhydrous K_2Co_3 (18.5 g, 133.897 mmol), 18-crown-6 (0.264 g) and 80 mL of anhydrous DMF were mixed in a 250 mL RB flask. It was refluxed at 120 °C for 48 hours. Then, DMF was evaporated completely and the crude mixture was subjected to silica gel column chromatography with 98:2 DCM: MeOH as eluent. Desired product **M5** was collected and washed with hexane to purify further to isolate in 73% yield (3.44 g). **^{1}H-NMR** (400 MHz, $CDCl_3$) δ in ppm: 8.42-8.40 (d, 4H), 8.26-8.24 (d, 2H), 8.08-8.06 (d, 2H), 7.73 (s, 2H), 7.13-7.11 (d, 4H), 4.26 (t, 4H), 3.92 (t, 4H), 3.78 (t, 4H), 3.76 (t, 4H), 3.73-3.66 (m, 12H), 3.38 (t, 4H). **^{13}C-NMR** (100 MHz, $CDCl_3$) δ in ppm: 160.07, 156.31, 145.98, 136.76, 132.29, 128.95, 127.52, 125.61, 119.35, 114.83, 70.87, 70.71, 70.66, 70.00, 69.72, 67.49, 50.65. **HR-MS (ESI$^+$):** Calcd. m/z for $C_{40}H_{47}N_8O_8[M+H]^+$ 767.3511, found 767.418.

(7) Macrocycle M6:

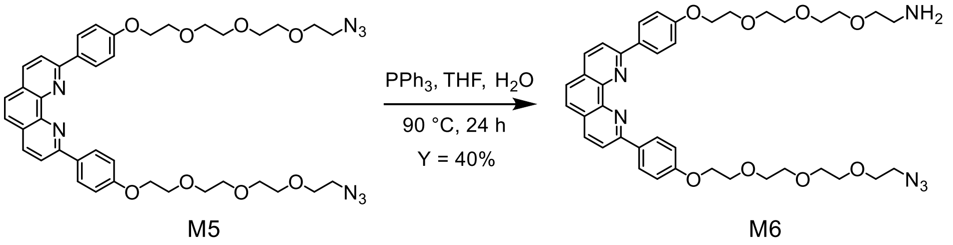

Monomer **M5** (0.5 g, 0.652 mmol), PPh_3 (0.171 g, 0.652 mmol) and H_2O (50 µL) were dissolved in 100 mL anhydrous THF under argon atmosphere. The reaction mixture refluxed at 90 °C for 24 hours. Later, it was evaporate to dryness under reduced pressure and purified by silica gel column chromatography using DCM: MeOH: Et_3N (90:10:0.5) as eluent to isolate desired mono-amine product, off-white thick liquid **M6** (0.178 g, yield 37%). Increasing the polarity to DCM: MeOH: Et_3N (90:10:3) results isolation of corresponding di-amine product in 40% yield (0.186 g). TLC visualization of both the amine products was carried out under UV-lamp as well as ninhydrin test. **^{1}H-NMR** (400 MHz, $CDCl_3$) δ in ppm: 8.40-8.38 (d, 4H), 8.24-8.22 (d, 2H), 8.06-8.04 (d, 2H), 7.71 (s, 2H), 7.13-7.11 (d, 4H), 4.24 (t, 4H), 3.91 (t, 4H), 3.76 (t, 4H), 3.74-3.61 (m, 12H), 3.59 (t, 2H), 3.52 (t, 2H), 3.37 (t, 2H), 2.86 (t, 2H), 2.80 (br, 2H). **^{13}C-NMR** (100

MHz, $CDCl_3$) δ in ppm: 160.00, 159.92, 156.26, 156.22, 145.85, 136.77, 132.22, 132.15, 128.93, 128.89, 127.46, 125.57, 119.36, 114.80, 71.93, 70.77, 70.71, 70.62, 70.58, 70.47, 70.43, 70.08, 69.93, 69.65, 67.41, 50.57, 41.24. **HR-MS (ESI$^+$):** Calcd. m/z for $C_{40}H_{49}N_6O_8[M+H]^+$ 741.3603, found 741.3651.

(8) Monomer M7:

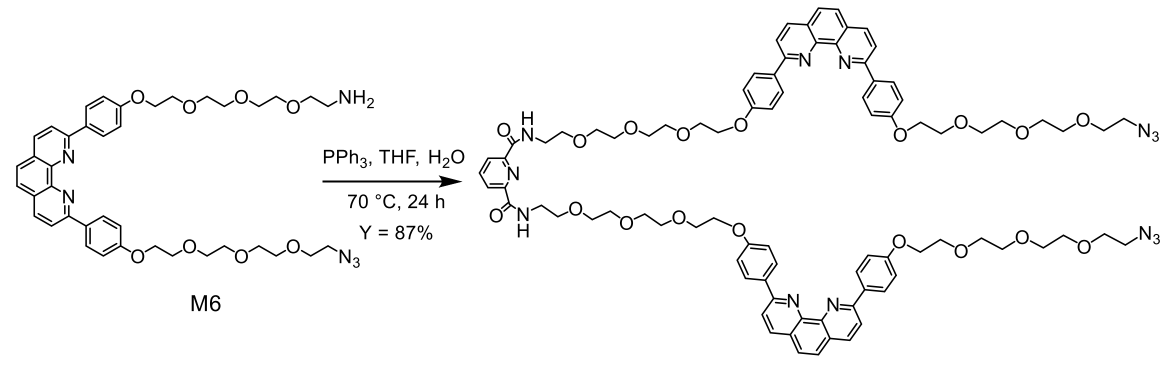

To a solution of monomer **M6** (0.65 g, 0.877 mmol) in 60 mL anhydrous DCM at 0 °C under argon atmosphere, Et_3N (0.146 mL, 1.05 mmol) was added. To this solution, 2,6-pyridinedicarbonyl dichloride (0.08 g, 0.45 equivalent, 0.394 mmol) dissolved in anhydrous DCM (20 mL) was added dropwise over 10 minutes at 0 °C. The final solution was stirred for 18 hours at RT before being concentrated in vacuum. The crude residue was purified by silica-gel column chromatography using DCM: MeOH, 97:3 as eluent to get 0.5 g of desired product **M7** with 80% yield. **^{1}H-NMR** (400 MHz, $CDCl_3$) δ in ppm: 9.01 (br, 2H), 8.39-8.34 (t, 8H), 8.28-8.26 (d, 2H), 8.23-8.18 (t, 4H), 8.05-8.00 (dd, 4H), 7.95-7.91 (t, 2H), 7.69 (s, 4H), 7.10-7.04 (dd, 4H), 4.23-4.21 (t, 4H), 4.18-4.16 (t, 4H), 3.90-3.88 (t, 4H), 3.85-3.82 (t, 4H), 3.75- 3.73 (t, 4H), 3.70-3.63 (m, 40H), 3.36-3.34 (t, 4H). **^{13}C-NMR** (100 MHz, $CDCl_3$) δ in ppm: 163.89, 159.99, 159.86, 156.24, 156.16, 148.76, 145.87, 138.55, 136.72, 132.23, 132.19, 128.89, 127.46, 125.55, 124.58, 119.33, 114.77, 114.70, 70.76, 70.66, 70.61, 70.57, 70.37, 70.15, 70.03, 69.91, 69.64, 69.59, 67.41, 50.58, 39.36. **HR-MS (ESI$^+$):** Calcd. m/z for $C_{87}H_{99}N_{13}O_{18}$ $[M+2H]^{2+}$ 806.8594, found 806.8376.

(9) Monomer M8:

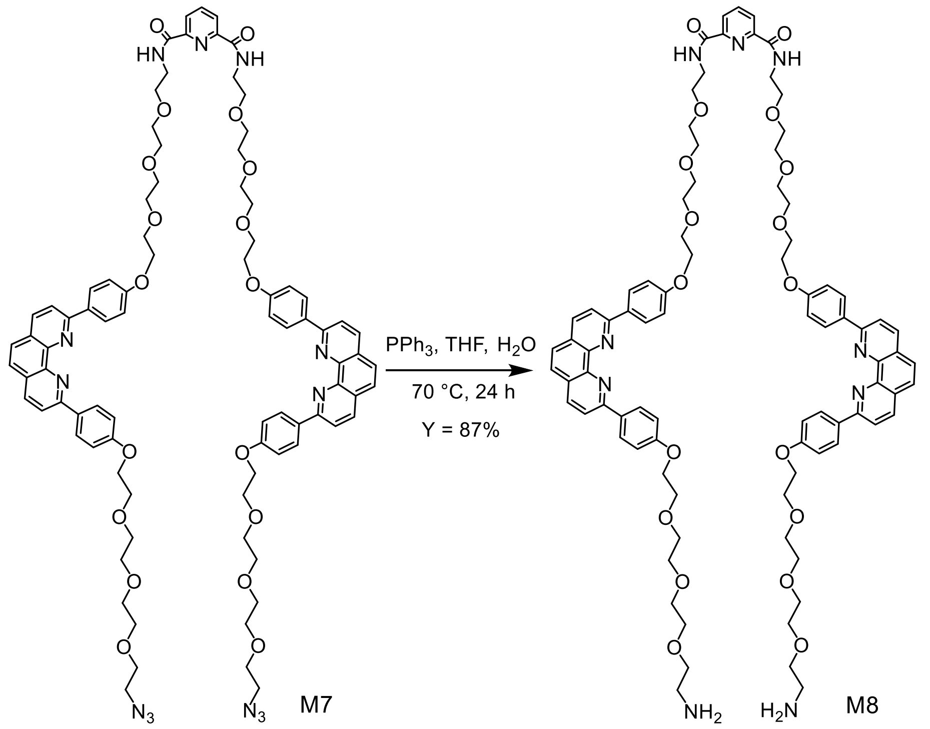

Monomer **M7** (0.595 g, 0.368 mmol), PPh_3 (0.483 g, 1.844 mmol) and H_2O (50 µL) were dissolved in 40 mL anhydrous THF under argon atmosphere. The reaction mixture refluxed at 90 °C for 24 hours. Later, it was evaporate to dryness under reduced pressure and purified by silica gel column chromatography using DCM: MeOH: Et_3N (90:10:0.6) as eluent to isolate desired di-amine product, off-white to cyan thick liquid **M8** (0.46 g, yield 80%). TLC visualization product was carried out under UV-lamp as well as ninhydrin test. **^{1}H-NMR** (400 MHz, $CDCl_3$) δ in ppm: 8.92 (br, 2H), 8.35 (t, 8H), 8.27-8.19 (m, 6H), 8.02 (t, 4H), 7.93 (t, 1H), 7.69 (s, 4H), 7.10-7.04 (dd, 8H), 4.22 (t, 4H), 4.16 (t, 4H), 3.88 (t, 4H), 3.82 (t, 4H), 3.73 (t, 4H), 3.68-3.58 (m, 32H), 3.53 (t, 4H), 3.48-3.43 (t, 4H), 3.24 (br, 4H), 2.88 (t, 4H). **^{13}C-NMR** (100 MHz, $CDCl_3$) δ in ppm: 163.87, 160.00, 159.93, 156.20, 148.86, 145.86, 138.54, 136.76, 132.21, 128.89, 127.47, 125.59, 124.62, 119.33, 114.84, 114.76, 71.55, 70.72, 70.69, 70.48, 70.43, 70.40, 70.12, 70.02, 69.86, 69.62, 67.49, 67.39, 41.13, 39.32. **HR-MS (ESI$^+$):** Calcd. m/z for $C_{87}H_{103}N_9O_{18}$ $[M+2H]^{2+}$ 780.8710, found 780.8674.

(10) Macrocycle MC-b:

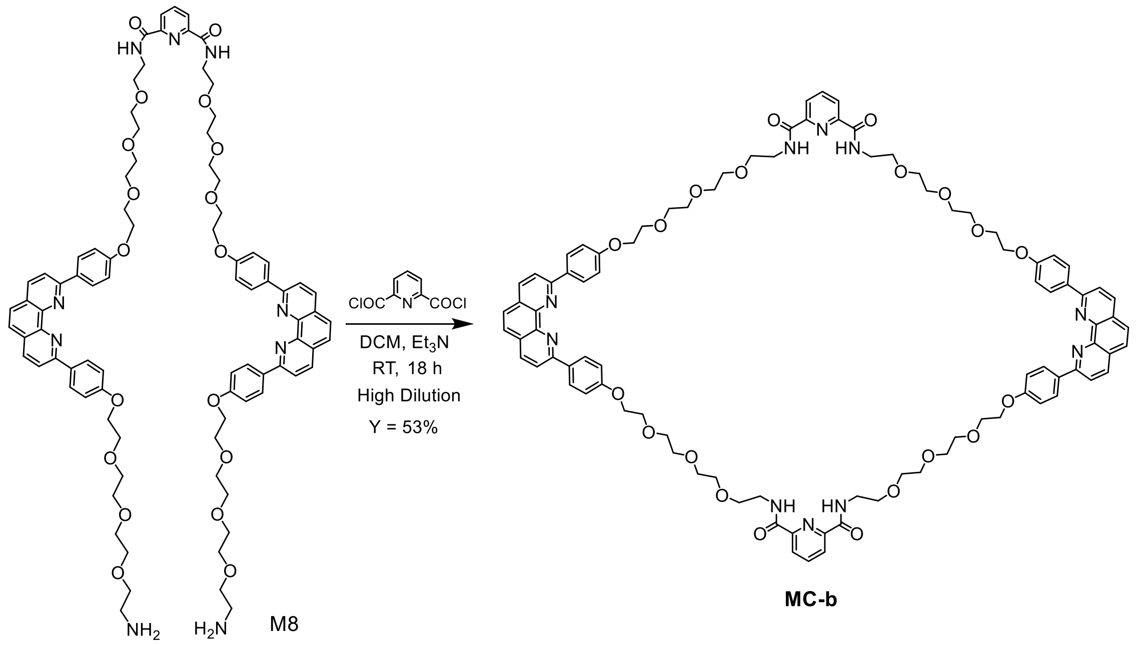

Macrocyclization was performed under high dilutions. To a 500 mL RB flask having monomer **M8** (0.46 g, 0.294 mmol) was added 276 mL of anhydrous DCM under argon atmosphere. This solution was then brought to 0 °C and to it triethylamine (0.091 mL, 0.657 mmol) was injected and stirred. To it, 2,6-pyridinedicarbonyl dichloride (0.06 g, 0.294 mmol) dissolved in 30 mL of anhydrous DCM was added dropwise over a 10 minutes at 0 °C. It was then stirred for 18 hours after bringing it to room temperature. The solvent was removed under reduced pressure and the crude was purified by silica gel column chromatography using DCM: MeOH (96.5:3.5) as eluent to get the desired product **MC-b** and Et_3NHCl salt mixture. A work up was performed with DCM and water to remove the Et_3NHCl salt into water layer. The DCM layer was concentrated and washed with hexane and EtOAc to get **MC-b** as cyan colored semi solid in 53% yield, 0.264 g. **^{1}H-NMR** (400 MHz, $CDCl_3$) δ in ppm: 9.09 (br, 4H), 8.34-8.32 (d, 8H), 8.28-8.26 (d, 4H), 8.18-8.16 (d, 4H), 8.00-7.98 (d, 4H), 7.94 (t, 2H), 7.65 (s, 4H), 7.06-7.03 (d, 8H), 4.17 (t, 8H), 3.84 (t, 8H), 3.71 (t, 8H), 3.66-3.65 (m, 40H). **^{13}C-NMR** (100 MHz, $CDCl_3$) δ in ppm: 163.93, 159.87, 156.09, 148.77, 145.80, 138.56, 136.70, 132.18, 128.90, 127.42, 125.53, 124.58, 119.28, 114.69, 70.66, 70.37, 70.19, 70.05, 69.61, 67.36, 39.38. **HR-MS (ESI$^+$):** Calcd. m/z for $C_{94}H_{103}N_{10}O_{20}$ $[M+H^+]^+$ 1692.738, found 1692.6906 and for $C_{94}H_{104}N_{10}O_{20}$ $[M+2H^+]^{2+}$ 846.8725, found 846.885.

(11) Figure of eight F8-Cu from MC-b

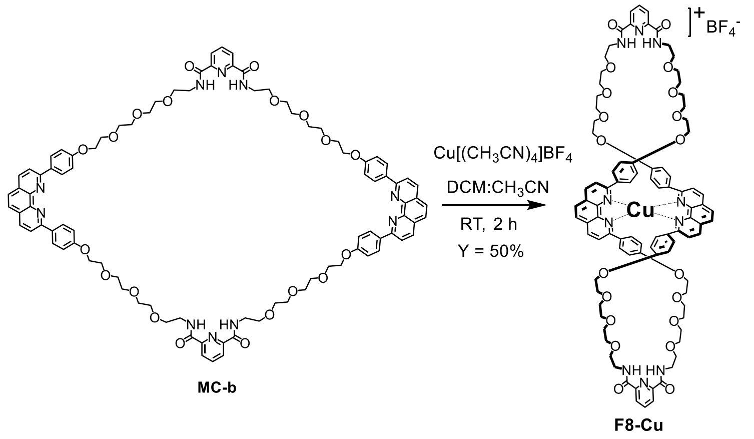

In a 100 ml RB flask, **MC-b** (0.260 g, 0.153 mmol) was transferred and 20 mL anhydrous DCM was added under argon atmosphere. To it, $Cu(CH_3CN)_4BF_4$ (0.0483 g, 0.153 mmol) dissolved in 20 mL CH_3CN was added under argon atmosphere. This solution was stirred at RT for 2 hours. It was concentrated in rotatory evaporator and subjected to silica gel column chromatography in 95:5 DCM: MeOH as eluent to isolate deep-red colored product. This product was washed 2 times with hexane to collect pure **F8-Cu** in 50% yield (0.134 g). **^{1}H-NMR** (400 MHz, $CDCl_3$) δ in ppm: 8.90 (br, 4H), 8.46-8.44 (d, 4H), 8.21-8.19 (d, 4H), 7.93 (s, 4H), 7.87-7.85 (d, 6H), 7.35-7.33 (d, 6H), 6.09-6.07 (d, 8H), 3.87-3.82 (t, 4H), 3.74-3.61 (m, 60H). **^{13}C-NMR** (100 MHz, $CDCl_3$) δ in ppm: 164.09, 159.17, 156.33, 148.89, 143.40, 138.30, 137.14, 131.34, 129.01, 127.71, 126.03, 124.57, 124.42, 113.39, 70.76, 70.55, 70.41, 69.93, 69.50, 69.43, 67.46, 38.94. **HR-MS (ESI$^+$):** Calcd. m/z for $C_{94}H_{102}CuN_{10}O_{20}Na[M^++Na^+]^{2+}$ 880.8234, found 846.8824.

(12) Demetalation of F8-Cu to form MC-b

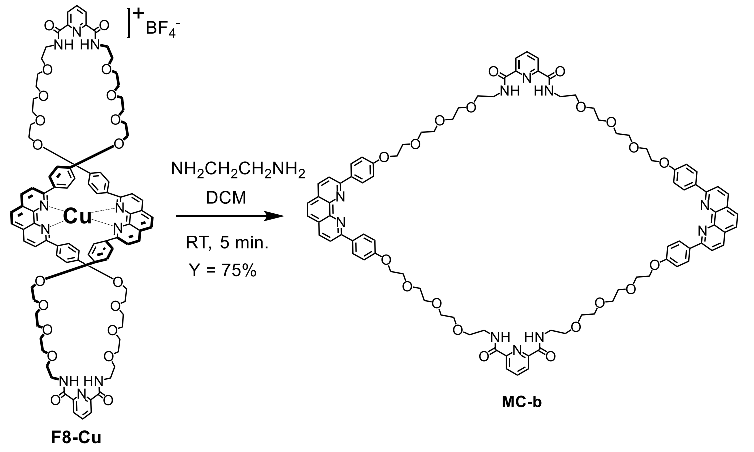

F8-Cu (0.078 g, 0.044 mmol) was dissolved in 40 ml of DCM and to it ethylene diamine was added in excess and stirred for 5 minutes. Immediate disappearance of deep-red color for the Cu(I) complex was observed and a colorless solution was formed. I was subjected to work up with water and DCM. DCM layer was collected, concentrated and subjected to silica gel column chromatography with 96:4 DCM: MeOH to isolate MC-b in impure form. It was washed with hexane 2 times to collect pure **MC-b** product in 75% yield (0.0564 g).

(13) Figure of eight F∞-Co from MC-b

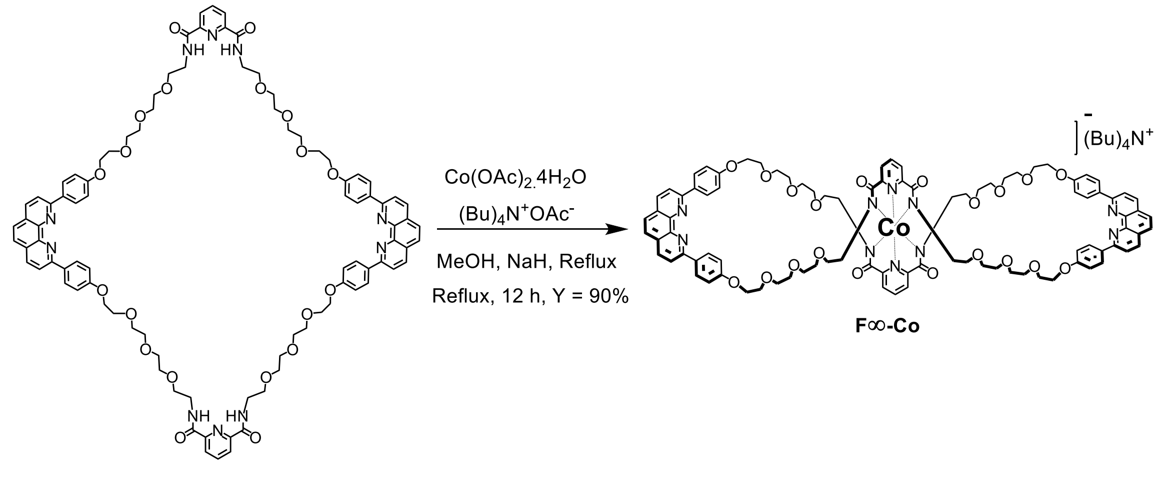

Cobalt metal complexation was performed according to reported literature procedure.[5] To a 100 mL two-neck round bottom flask **MC-b** (0.475 g, 0.28 mmol), $Co(OAc)_2.(H_2O)_4$ (0.069 g, 0.28 mmol) and Bu_4NOAc (0.084 g, 0.28 mmol) was transferred and it was purged with anhydrous methanol (25 mL) under inert atmosphere. The mixture was refluxed at 80 °C for 1 hours under inert atmosphere to obtain a clear pale pink solution. In another RB flask, sodium methoxide was prepared (by the slow addition of 25 mL anhydrous methanol into NaH (1 g) at 0 °C and injected into the reaction mixture at reflux conditions. This results a color change to deep-red. Later, on exposure to air the solution turned to green and it was further refluxed for 12 hours. Later solvent was evaporated to dryness and the residue was subjected to SiO_2 column chromatography using DCM: MeOH (92:8 v/v) as eluent to collect green colored product **F∞-Co**. It was further purified by washing with Hexane and EtOAc two times to result pure **F∞-Co** in 90% yield (0.441 g). **^{1}H-NMR** (400 MHz, $CDCl_3$) δ in ppm: 8.41-8.39 (d, 8H), 8.23-8.21 (d, 6H), 8.05-8.03 (d, 4H), 7.81 (br, 4H), 7.70 (br, 4H), 7.13-7.10 (d, 8H), 4.21 (br, 8H), 3.83 (br, 8H), 3.62 (br, 8H), 3.52 (br, 8H), 3.37 (br, 8H), 3.20 (br, 8H), 2.75 (br, 8H), 2.23 (br, 4H), 1.98 (br, 4H). **^{13}C-NMR** (100 MHz, $CDCl_3$) δ in ppm: 168.19, 160.00, 156.65,

156.10, 145.89, 140.35, 136.77, 132.29, 128.93, 127.48, 125.59, 123.47, 119.25, 114.96, 70.50, 70.10, 69.90, 69.54, 67.44, 42.76. **HR-MS (ESI⁺):** Calcd. m/z for $C_{94}H_{100}CoN_{10}O_{20}Na[M^- +2H^+ +Na^+]^{2+}$ 885.8190, found 885.8425 and for $C_{94}H_{100}CoN_{10}O_{20}Na_2[M^- +2H^+ +2Na^+]^{3+}$ 598.2079, found 598.2097.

(14) Demetalation of F∞-Co from MC-b

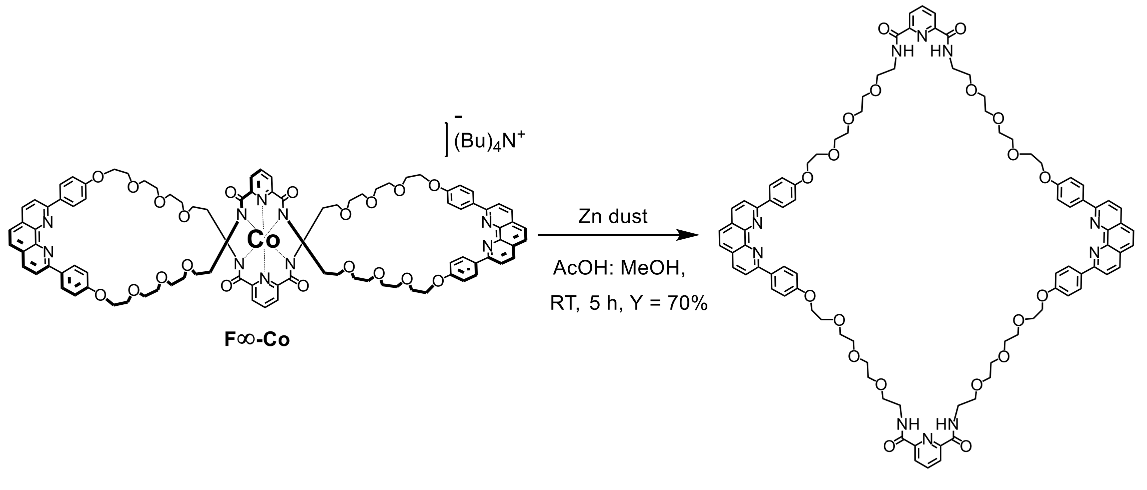

Removal of cobalt metal from **F∞-Co** was done according to reported literature procedure.[6] 0.1 g of **F∞-Co** was transferred to a RB flask and to it anhydrous MeOH and CH_3COOH 20 ml each was added followed by addition of activated Zinc 0.6 g. The reaction mixture was stirred for 5 hours in open air. Partial disappearance of green color from the reaction mixture can be used as an indication for completion of reaction. Later, solvent was removed to dryness using rotatory evaporator and 30 mL of $CHCl_3$ was added. To it 1:1 mixture of 12.5 mL of 17.5% ammonia solution and 12.5 mL of saturated Na_4EDTA was added. The mixture was stirred for 30 minutes and work up was performed with $CHCl_3$ 3 times. Organic layer was collected and again work up was performed using brine solution. Later organic layer was collected, dried with anhydrous Na_2SO4, evaporated to dryness and purified by column chromatography using DCM: MeOH in 96:4 as eluent to isolate **MC-b** in 70% yield (0.0745 g).).

12.5 mL of saturated Na_4EDTA was prepared by mixing 5 g of EDTA with 2.73 g of NaOH and making the volume up to 12.5 mL with water and stirring it well. Activated Zinc was prepared by taking 10 g of Zinc powder in a RB flask and washing it with 2N HCl three times followed by washing with DI water, ethanol, acetone, diethyl ether two times each and drying in hot air oven at 100 °C for 15 minutes.

(d) NMR Spectra:

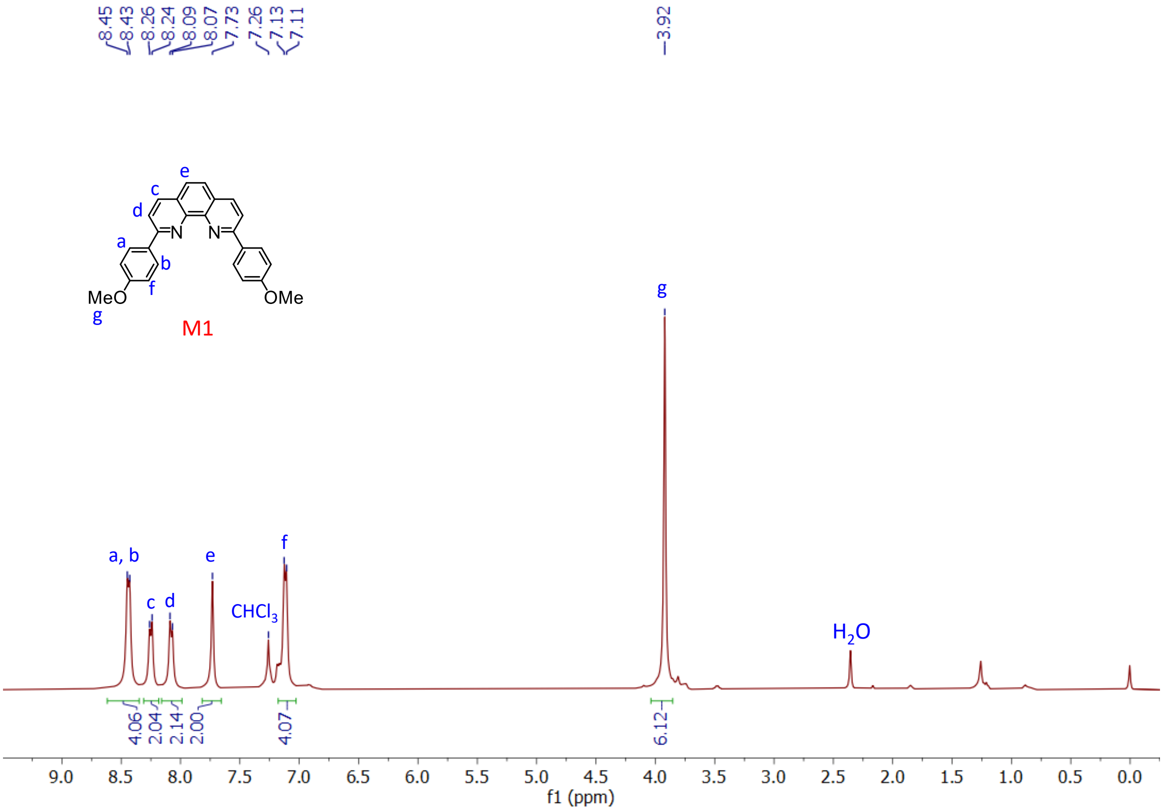

Figure S1. ^{1}H-NMR spectra of monomer M1 in $CDCl_3$ (400 MHz).

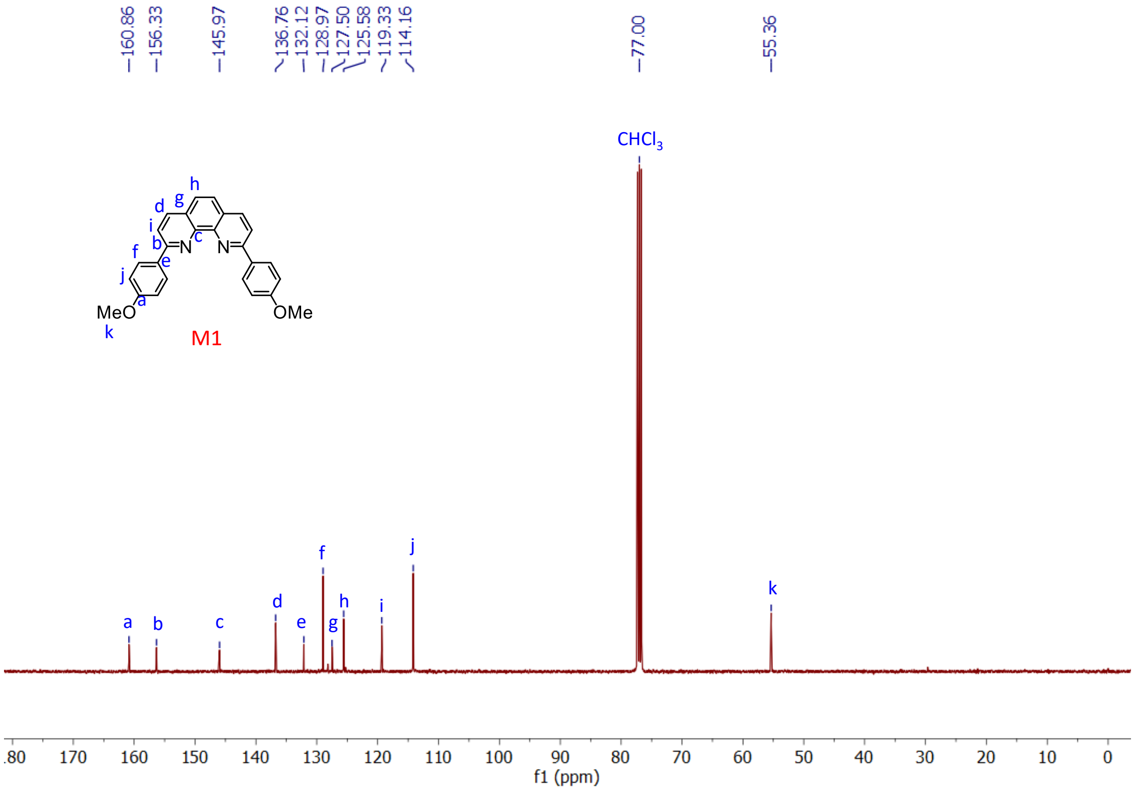

Figure S2. ^{13}C-NMR spectra of monomer M1 in $CDCl_3$ (400 MHz).

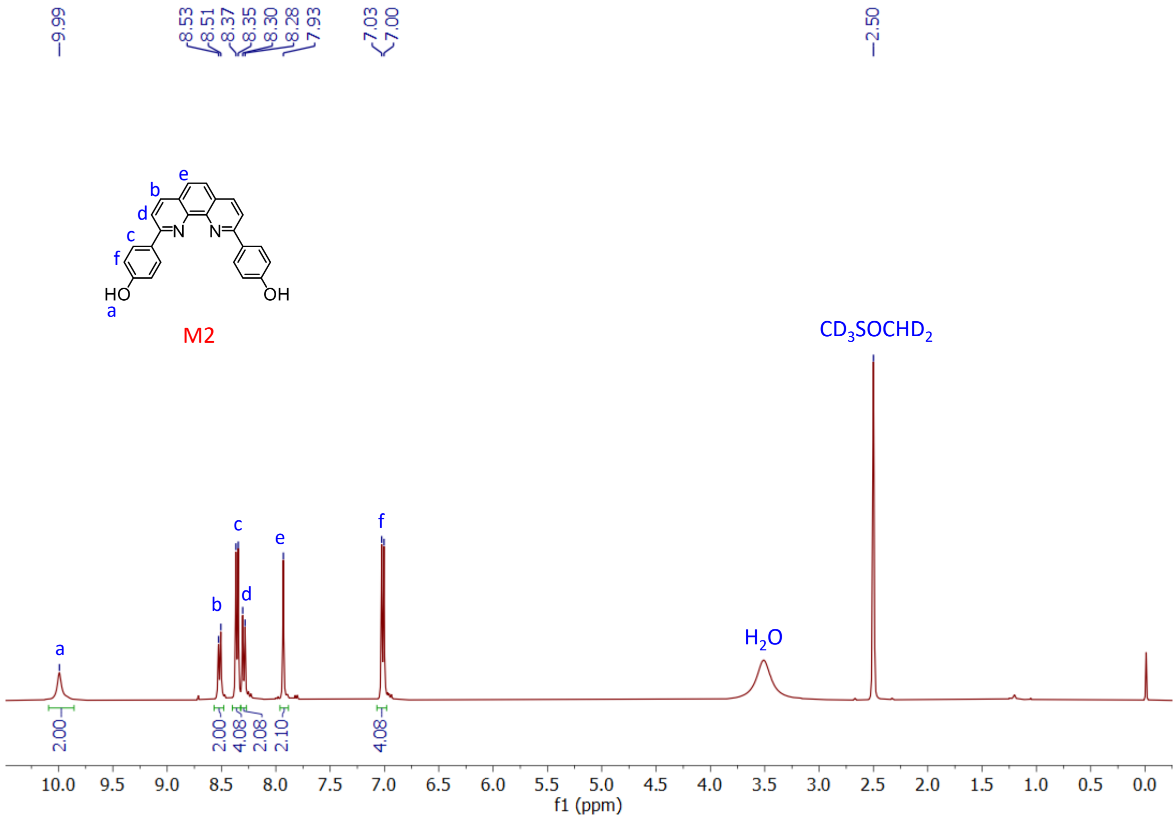

Figure S3. ^{1}H-NMR spectra of monomer M2 (Phen-OH) in DMSO-D6 (400 MHz).

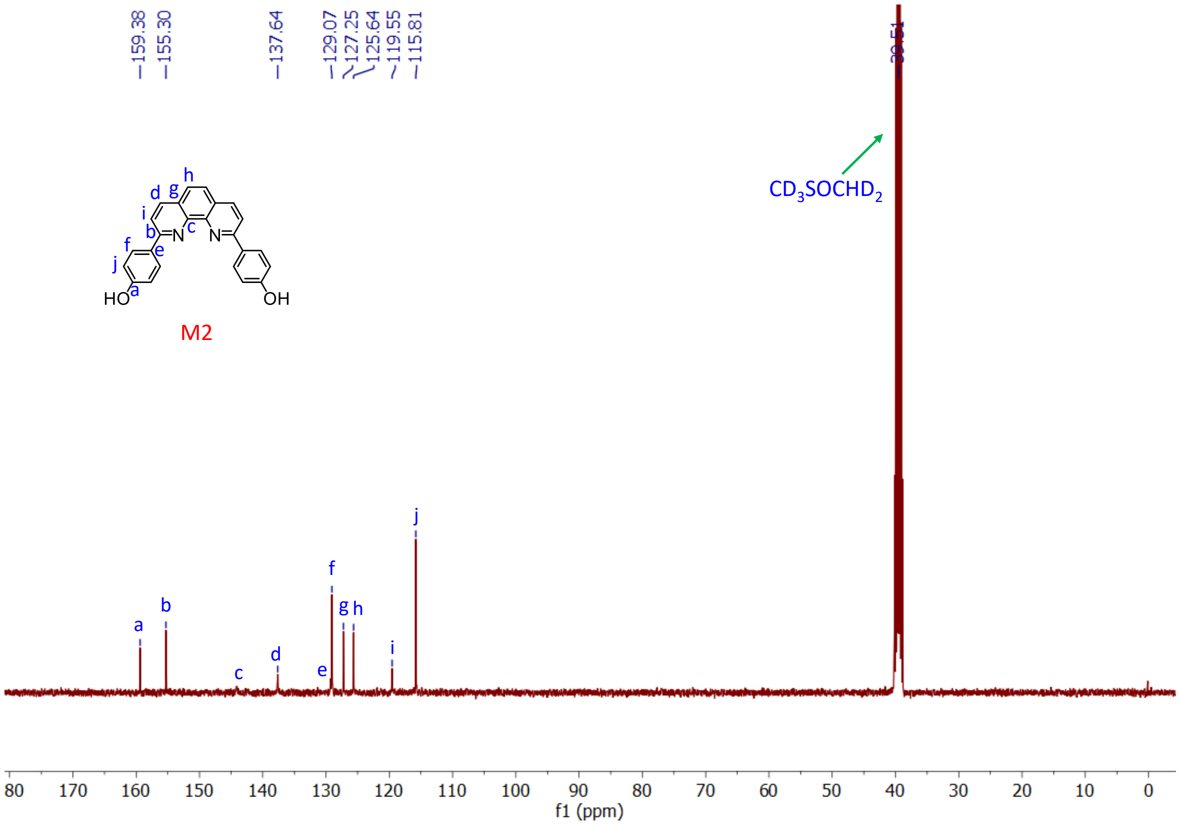

Figure S4. ^{13}C-NMR spectra of monomer M2 (Phen-OH) in DMSO-D6 (400 MHz).

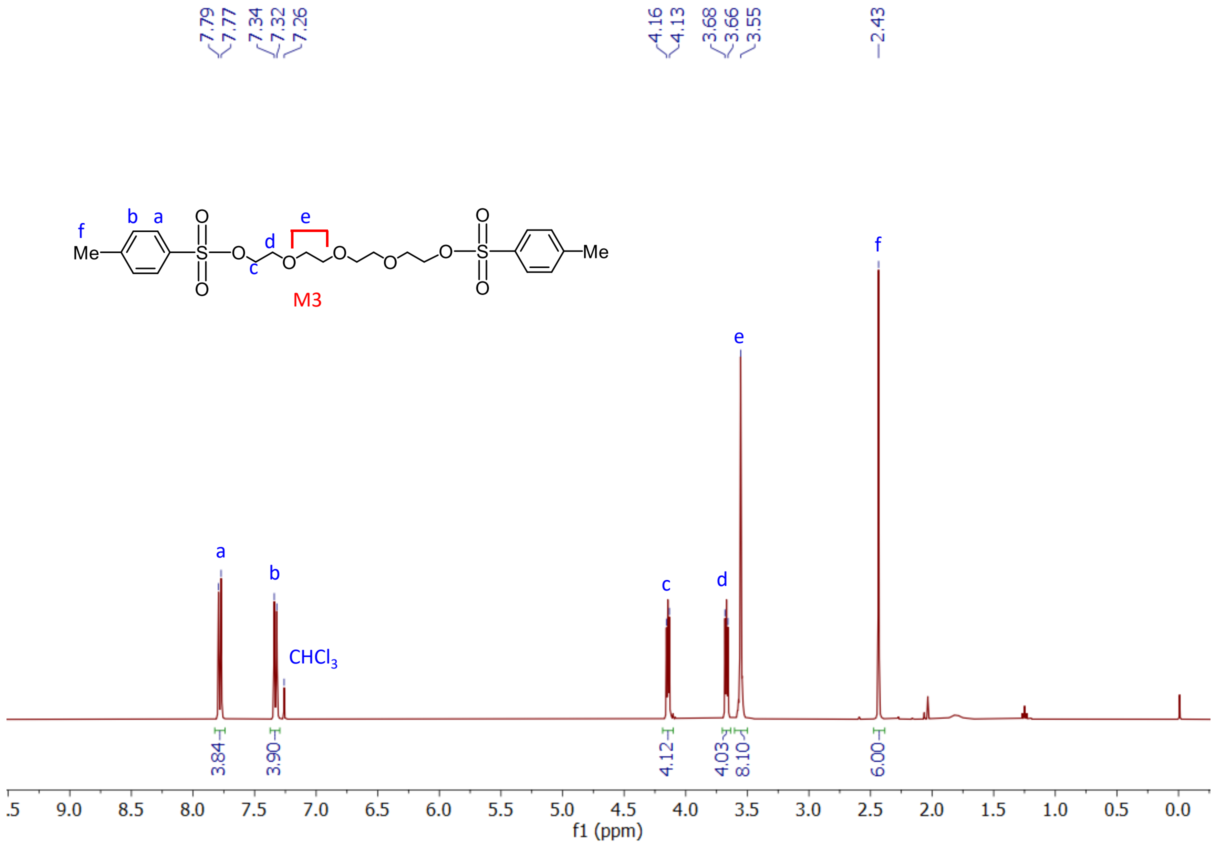

Figure S5. ^{1}H-NMR spectra of monomer M3 in $CDCl_3$ (400 MHz).

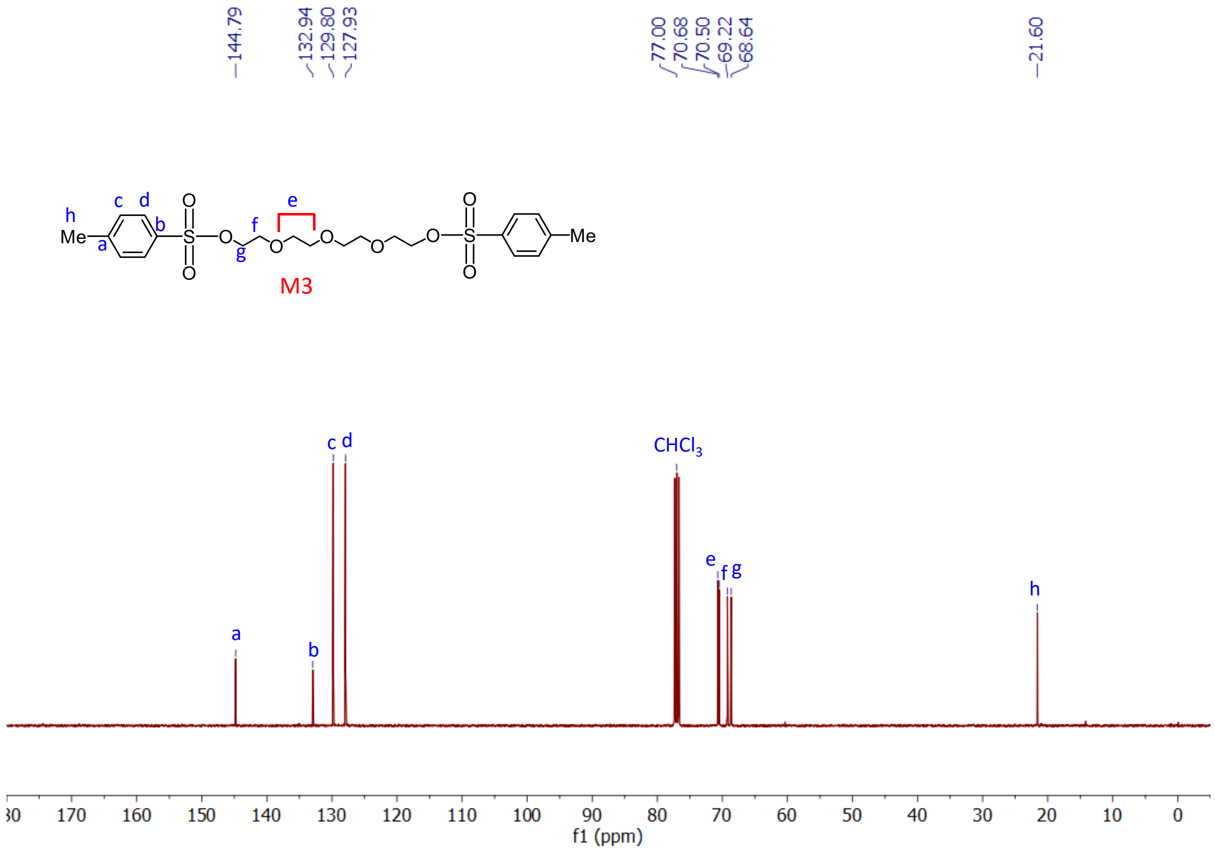

Figure S6. ^{13}C-NMR spectra of monomer M3 in $CDCl_3$ (400 MHz).

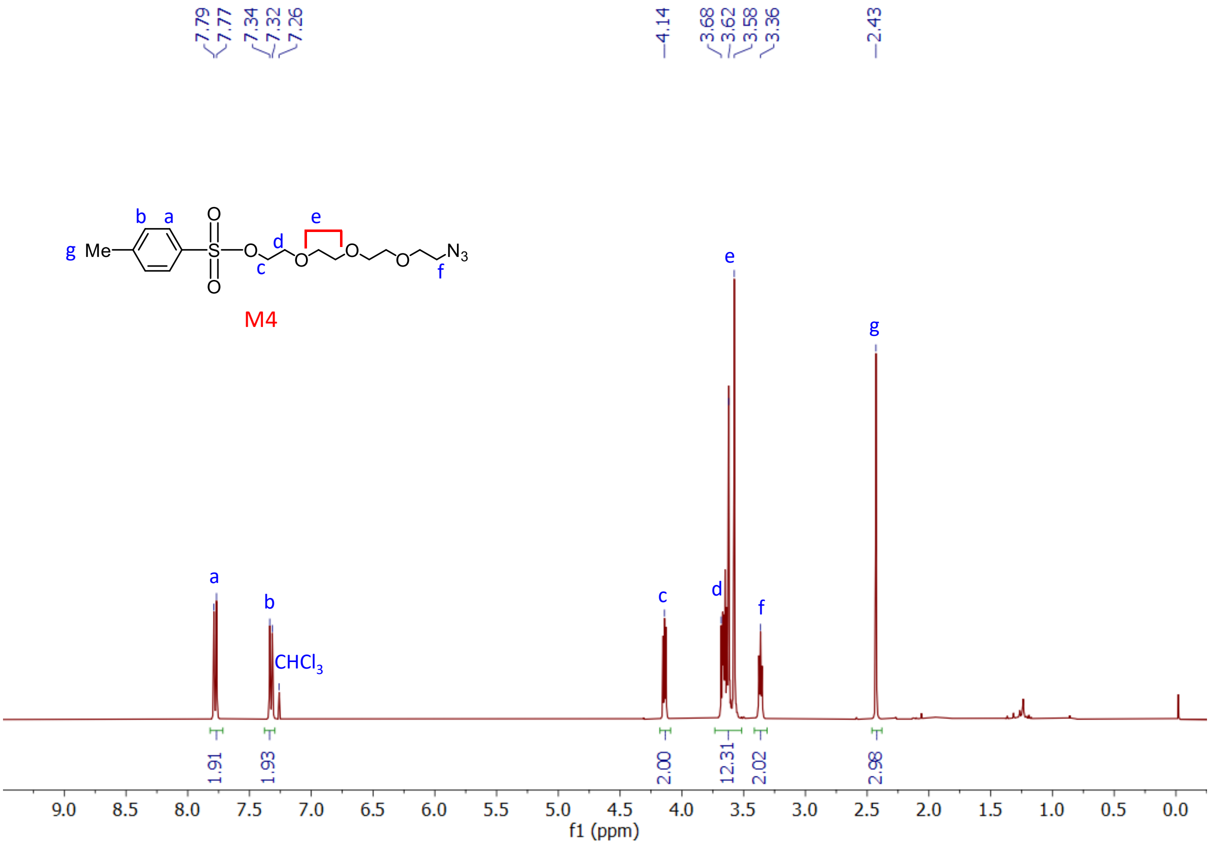

Figure S7. ^{1}H-NMR spectra of monomer M4 in $CDCl_3$ (400 MHz).

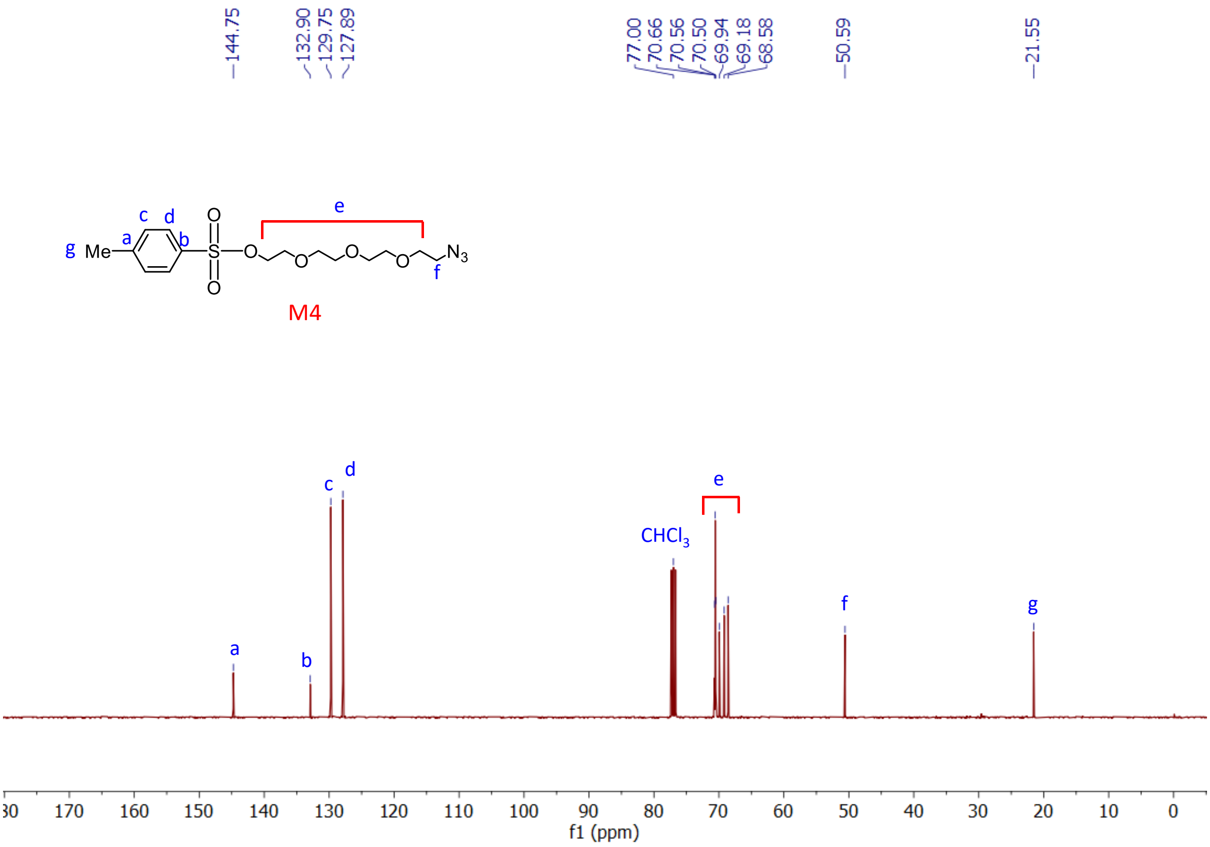

Figure S8. ^{13}C-NMR spectra of monomer M4 $CDCl_3$ (400 MHz).

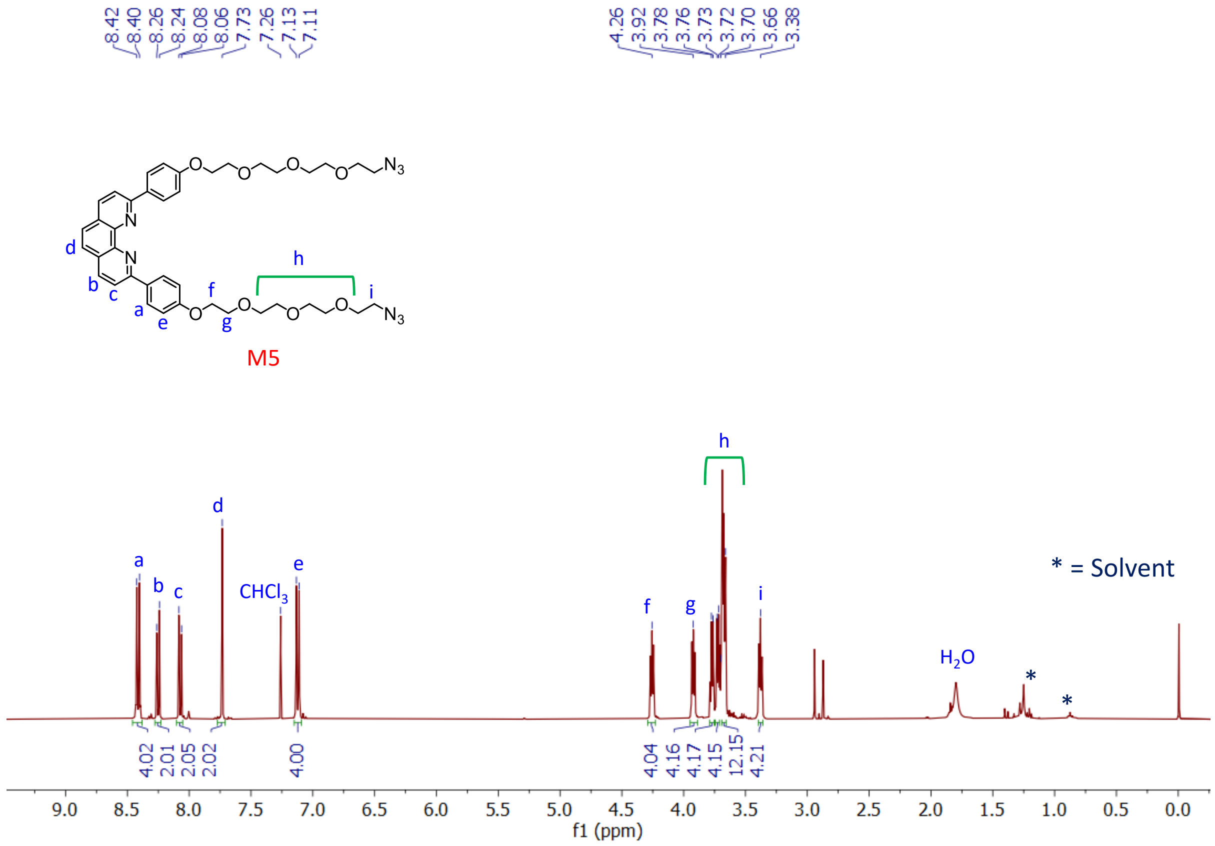

Figure S9. ^{1}H-NMR spectra of monomer M5 in $CDCl_3$ (400 MHz).

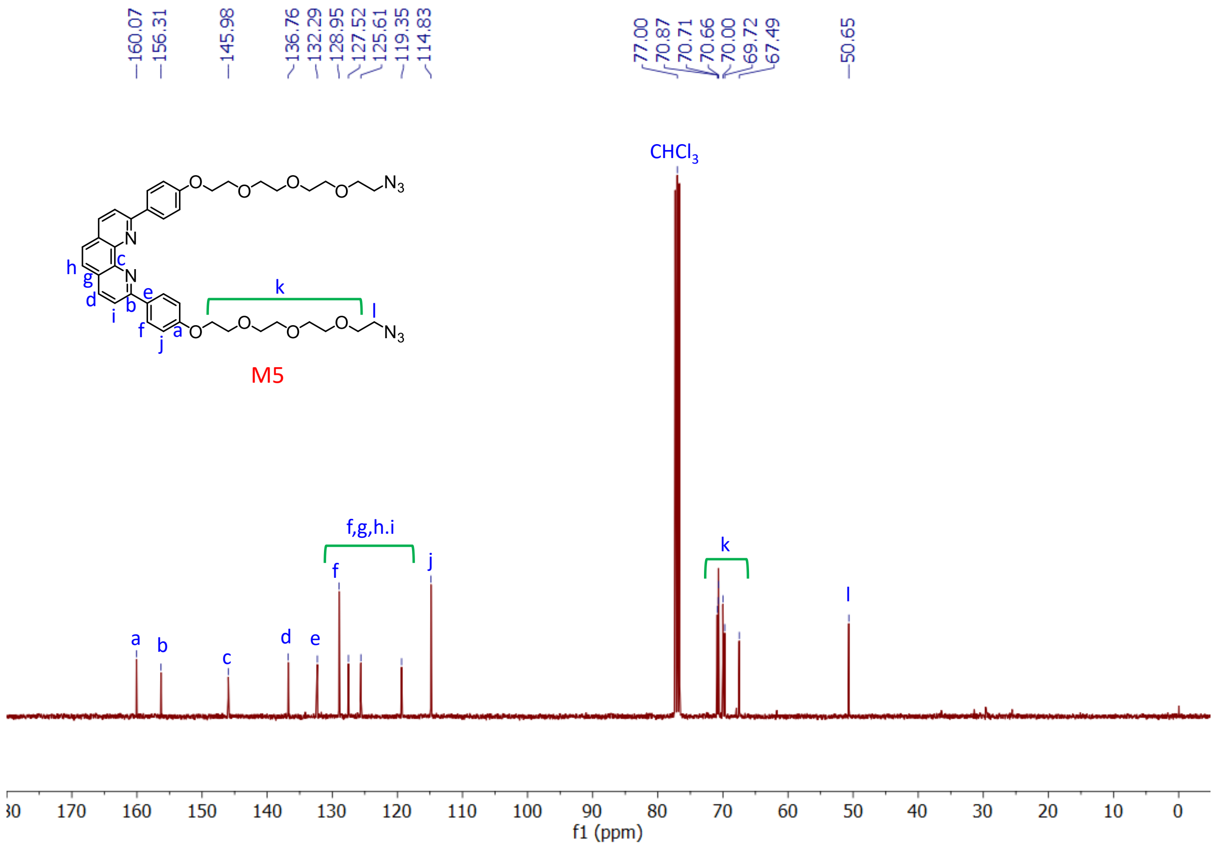

Figure S10. ^{13}C-NMR spectra of monomer M5 in $CDCl_3$ (400 MHz).

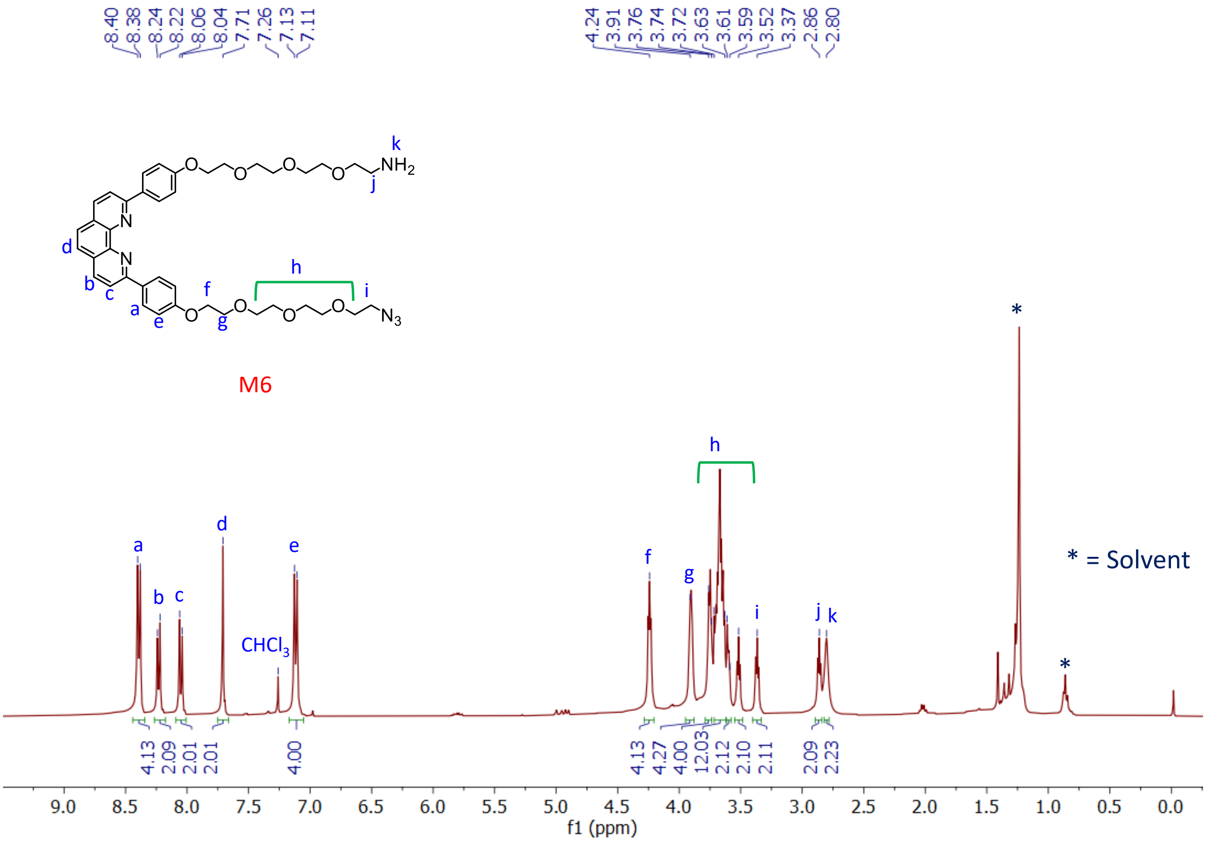

Figure S11. ^{1}H-NMR spectra of monomer M6 in $CDCl_3$ (400 MHz).

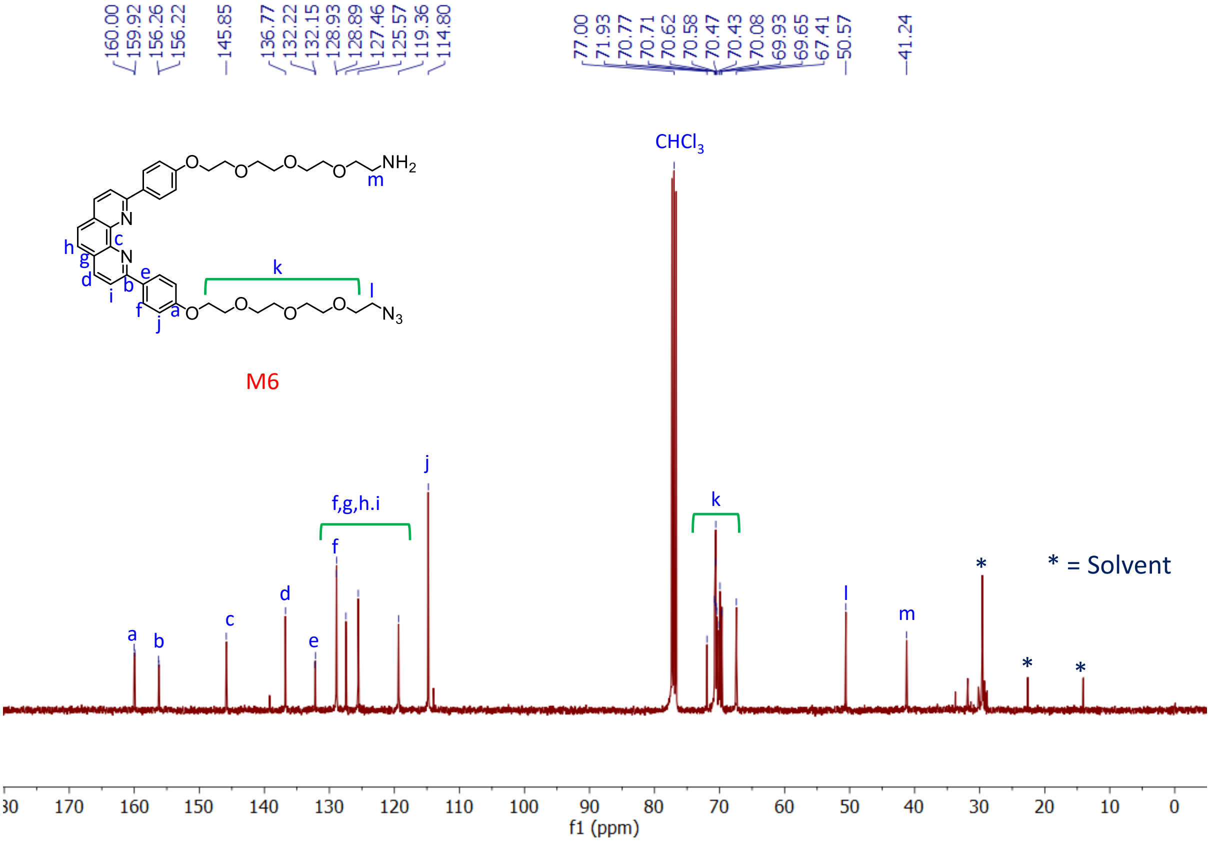

Figure S12. ^{13}C-NMR spectra of monomer M6 in $CDCl_3$ (400 MHz).

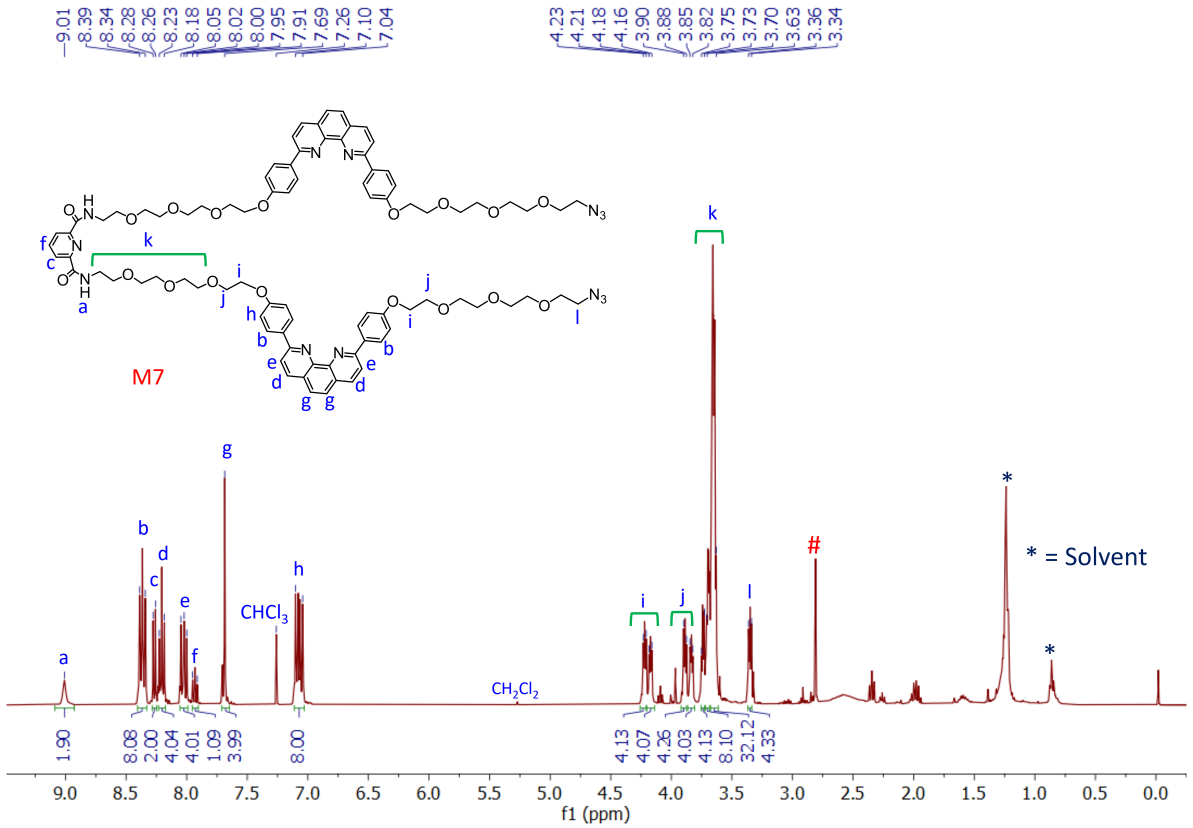

Figure S13. ^{1}H-NMR spectra of di-azide terminal monomer M7 in $CDCl_3$ (400 MHz).

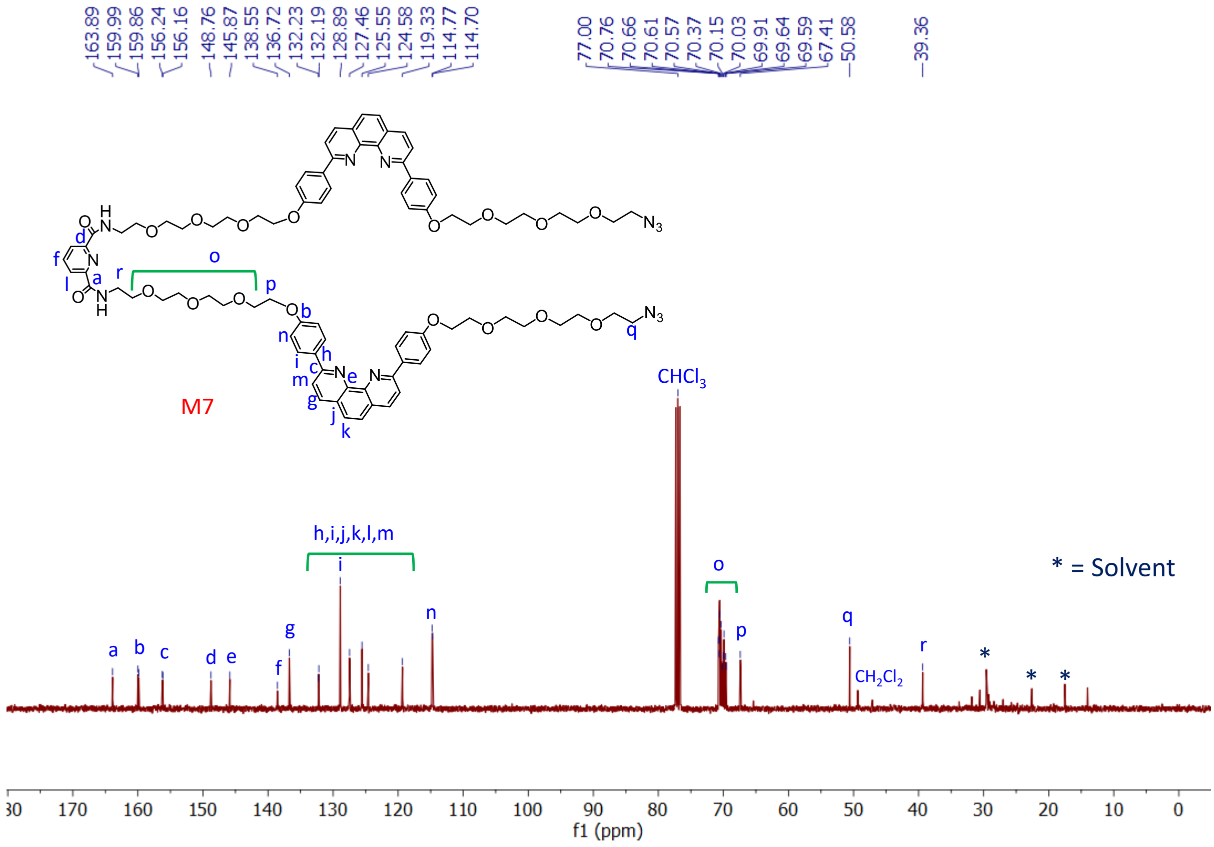

Figure S14. ^{13}C-NMR spectra of di-azide terminal monomer M7 in $CDCl_3$ (400 MHz).

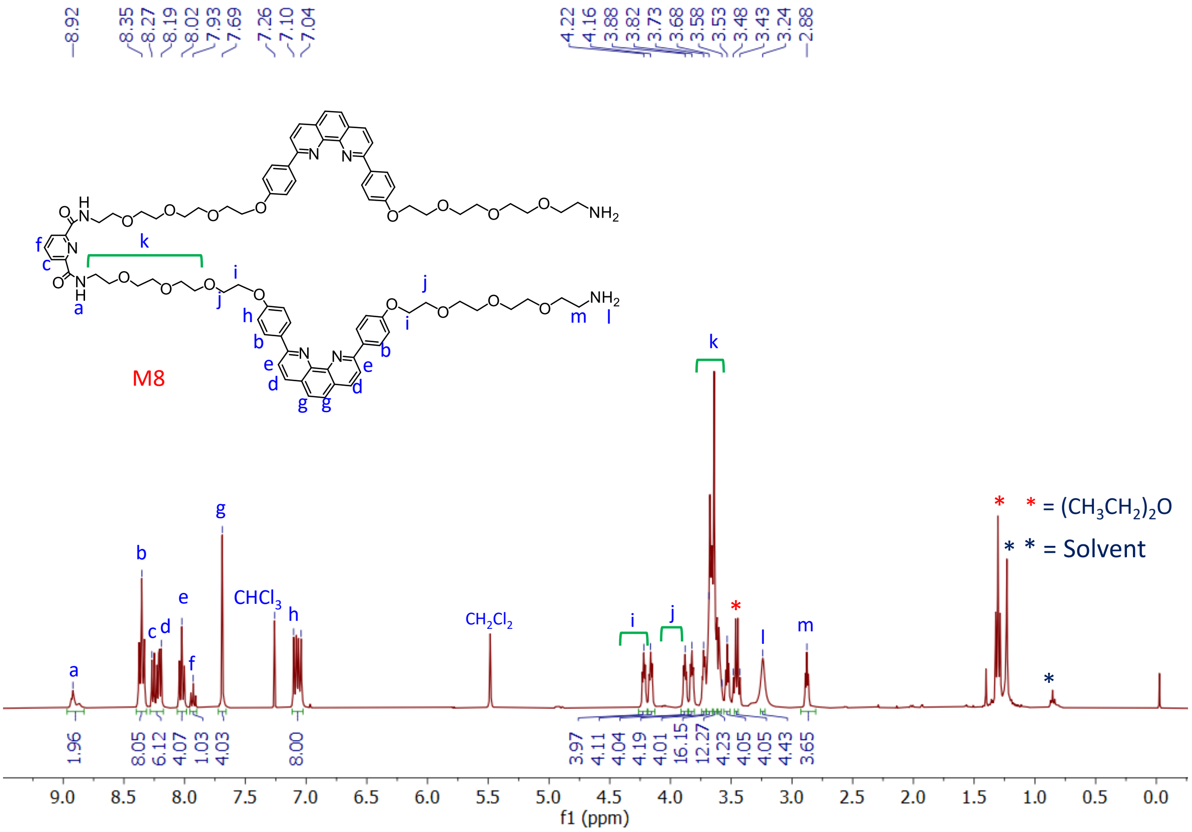

Figure S15. ^{1}H-NMR spectra of di-amine monomer M8 in $CDCl_3$ (400 MHz).

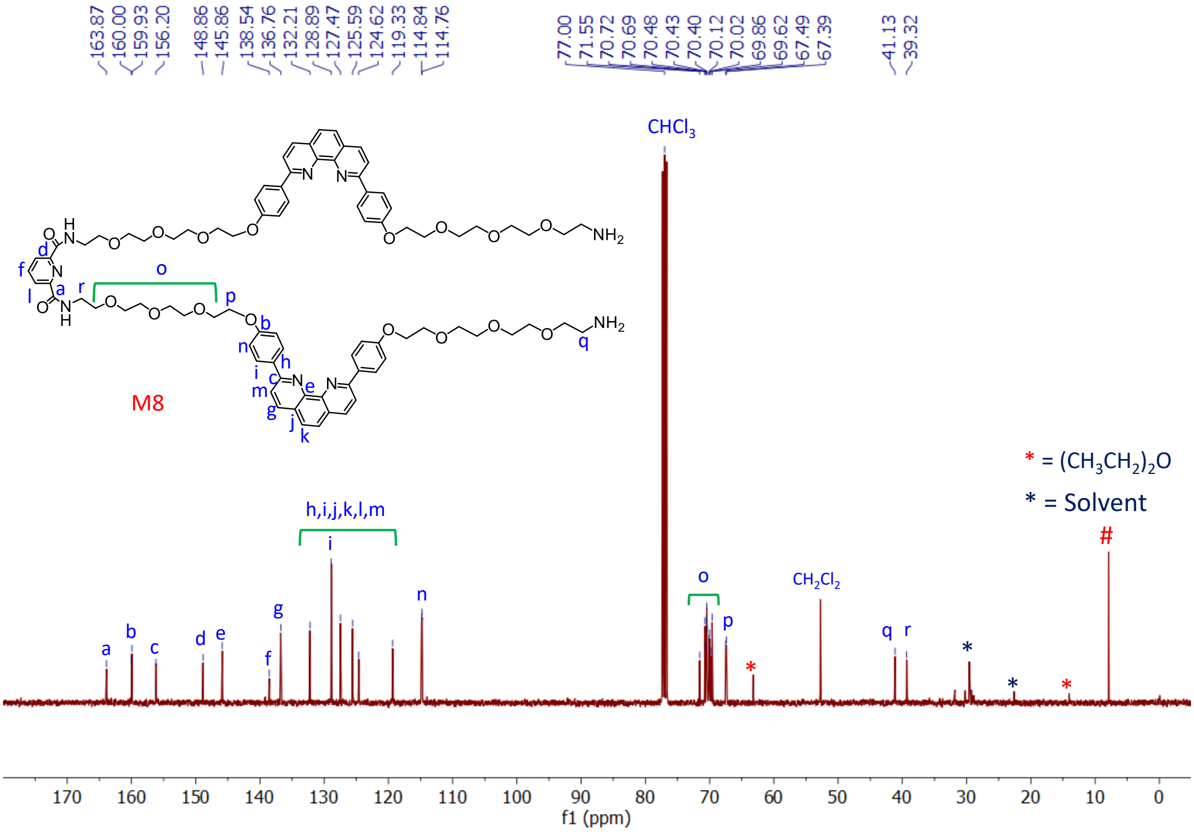

Figure S16. ^{13}C-NMR spectra of di-amine monomer M8 in $CDCl_3$ (400 MHz).

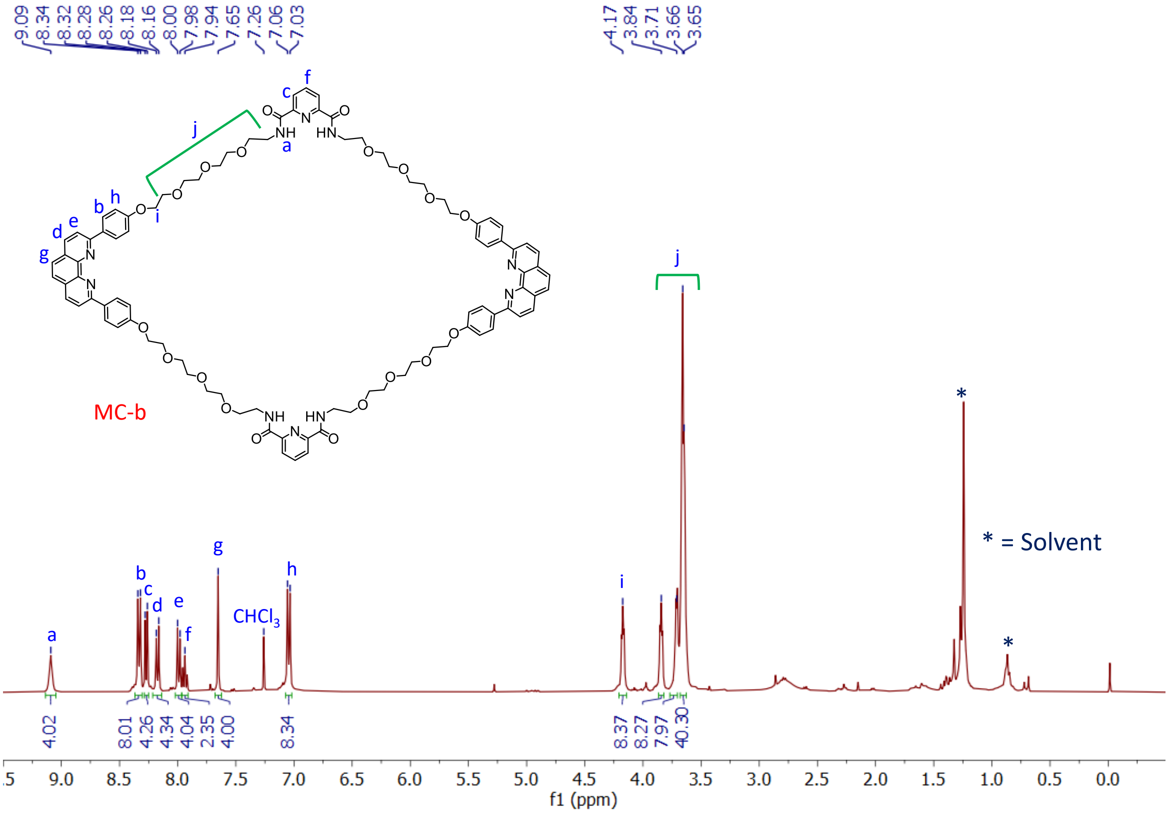

Figure S17. ^{1}H-NMR spectra of bi-modal macrocycle MC-b in $CDCl_3$ (400 MHz).

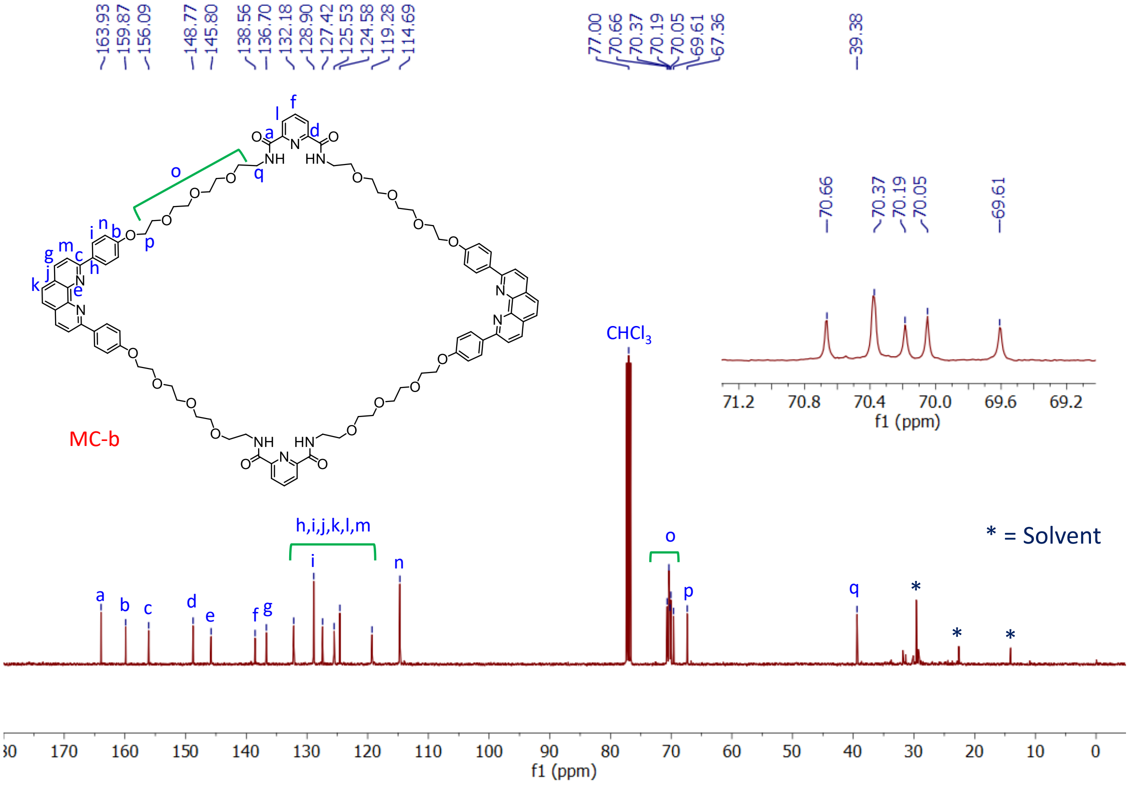

Figure S18. ^{13}C-NMR spectra of bi-modal macrocycle MC-b in $CDCl_3$ (400 MHz).

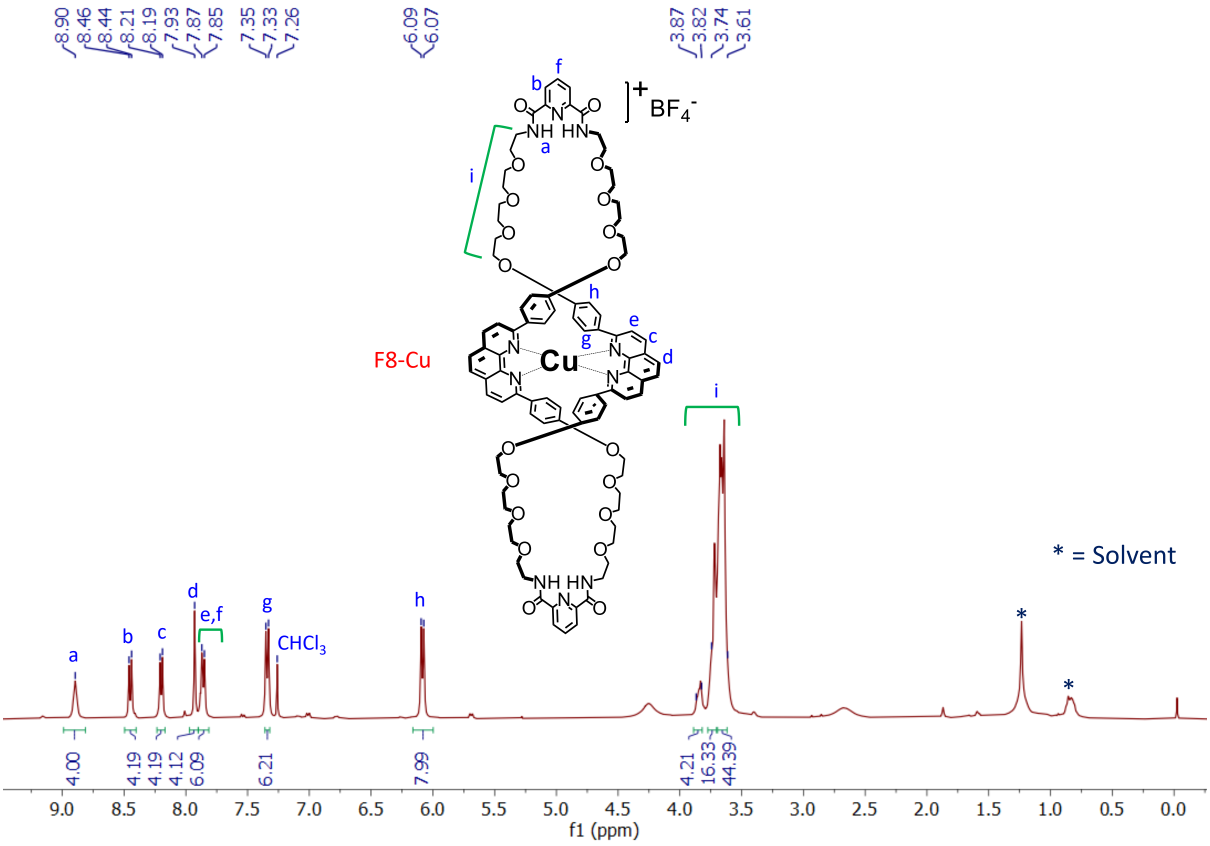

Figure S19. ^{1}H-NMR spectra of F8-Cu complex in $CDCl_3$ (400 MHz).

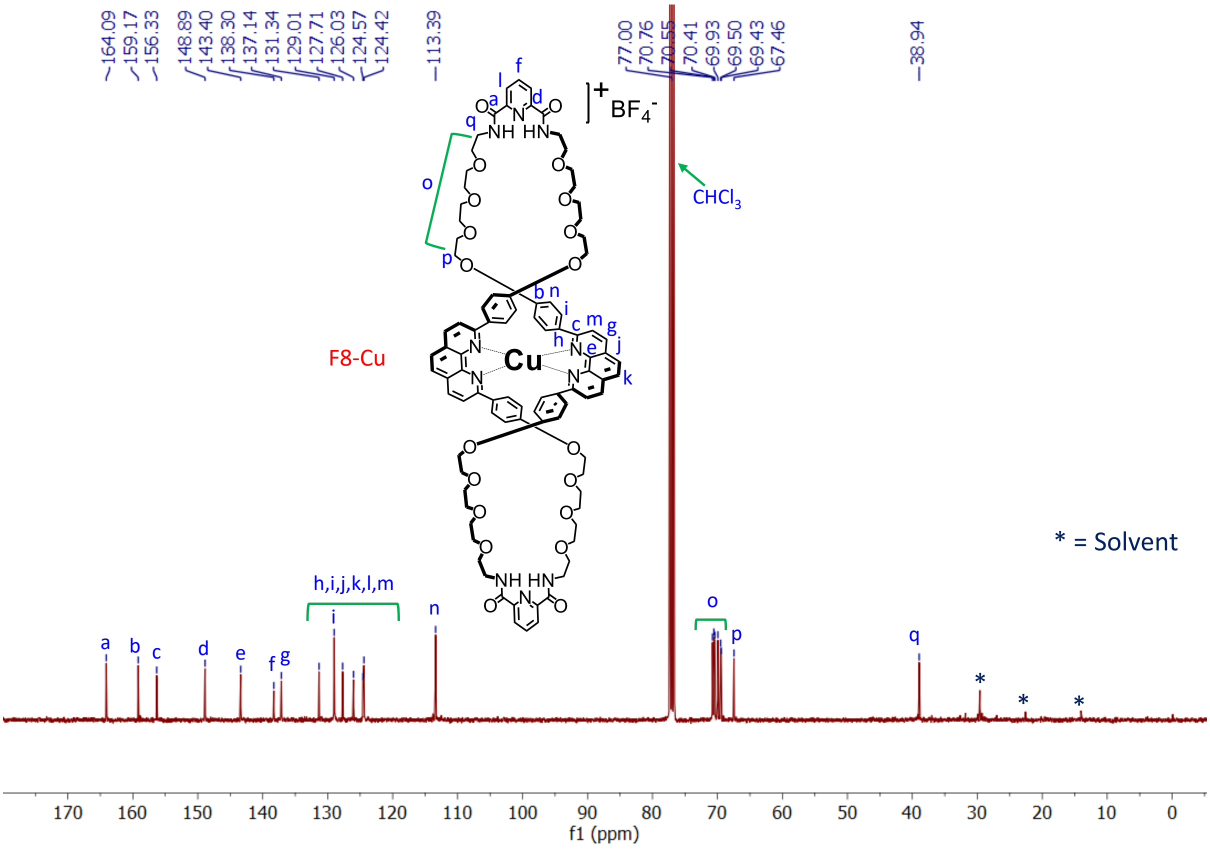

Figure S20. ^{13}C-NMR spectra of F8-Cu complex in $CDCl_3$ (400 MHz).

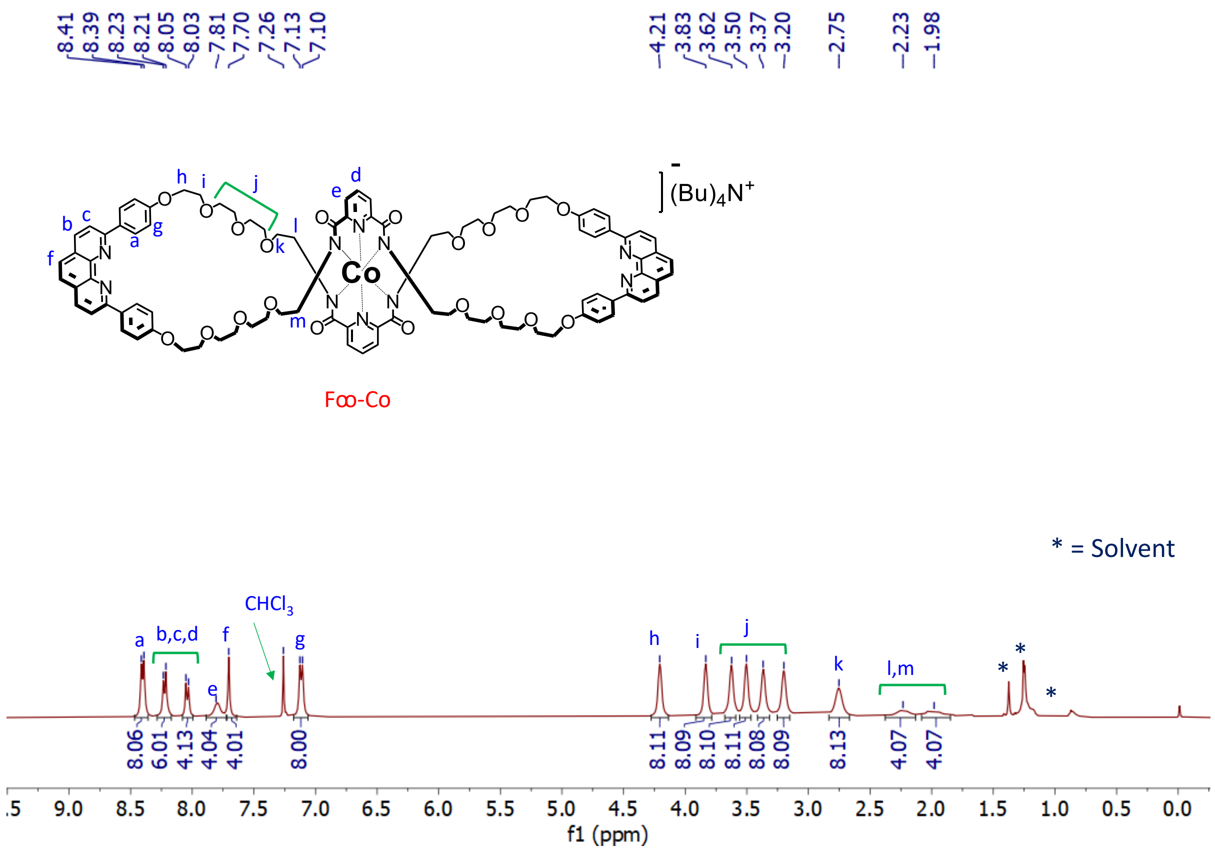

Figure S21. ^{1}H-NMR spectra of F∞-Co complex in $CDCl_3$ (400 MHz).

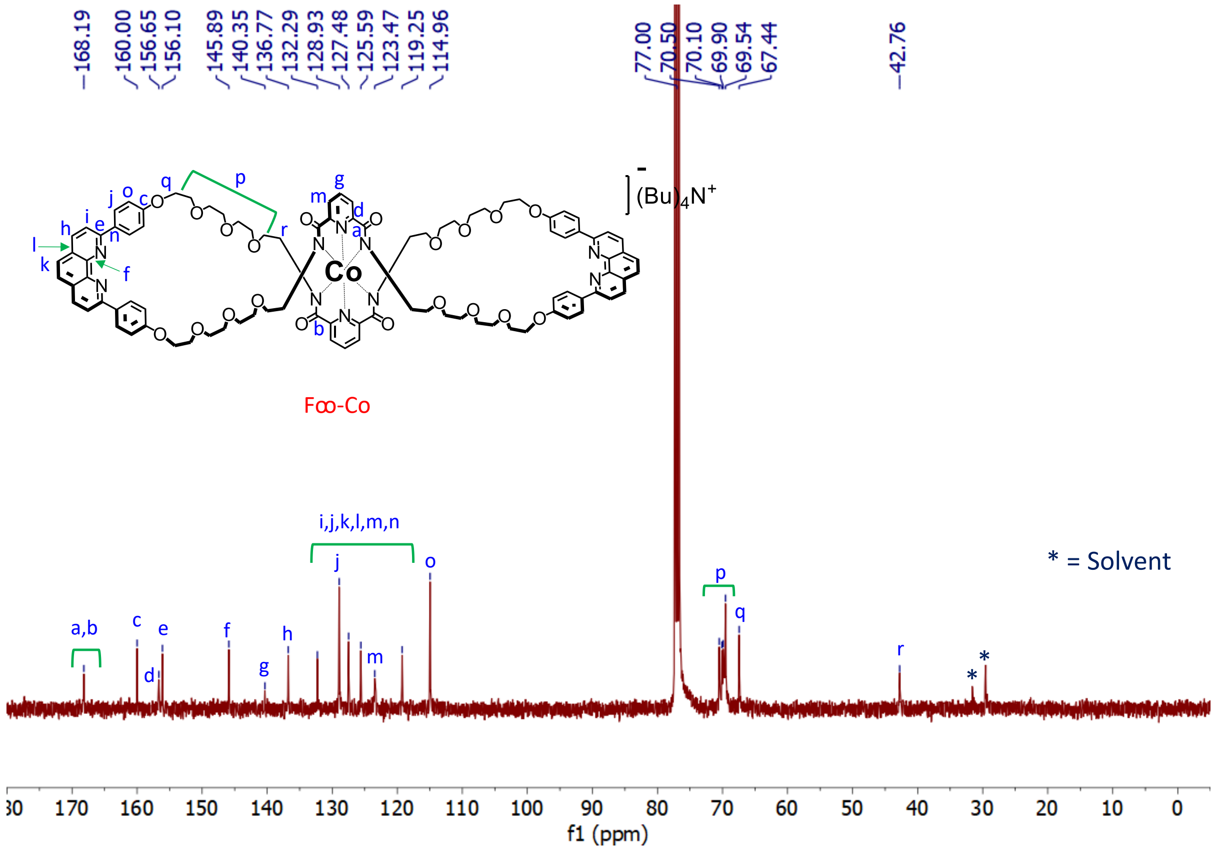

Figure S22. ^{13}C-NMR spectra of F8-Co complex in $CDCl_3$ (400 MHz).

(e) Mass Spectra:

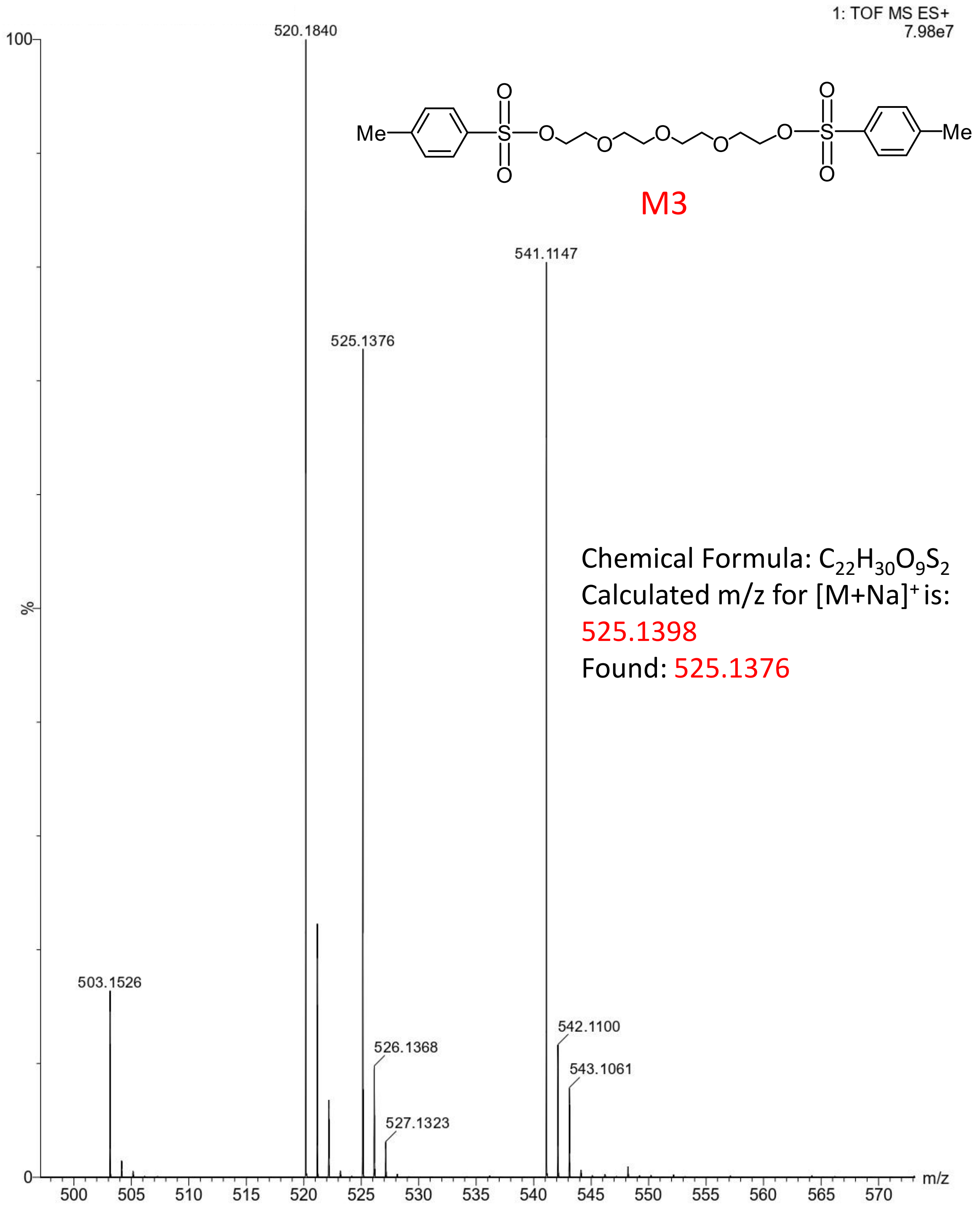

Figure S23. Mass spectrum (ESI^+) analysis for monomer M3.

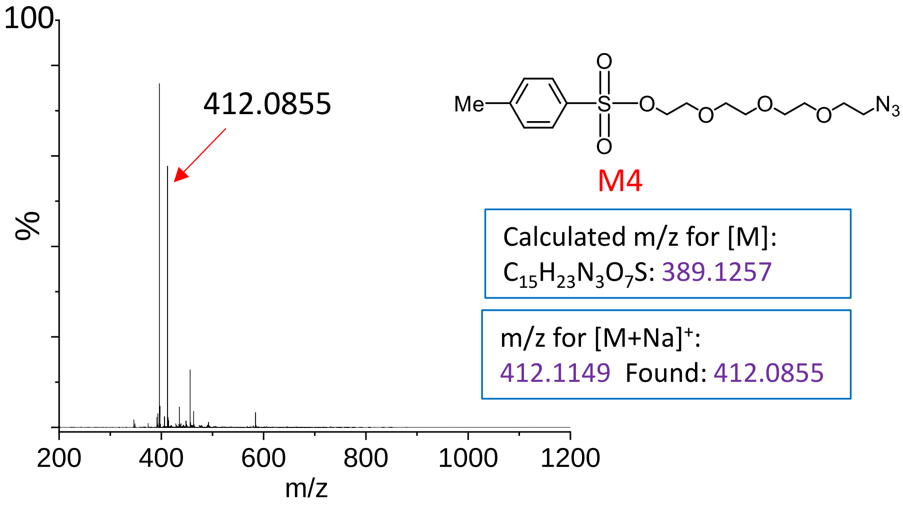

Figure S24. Mass spectrum (ESI+) analysis for monomer M4.

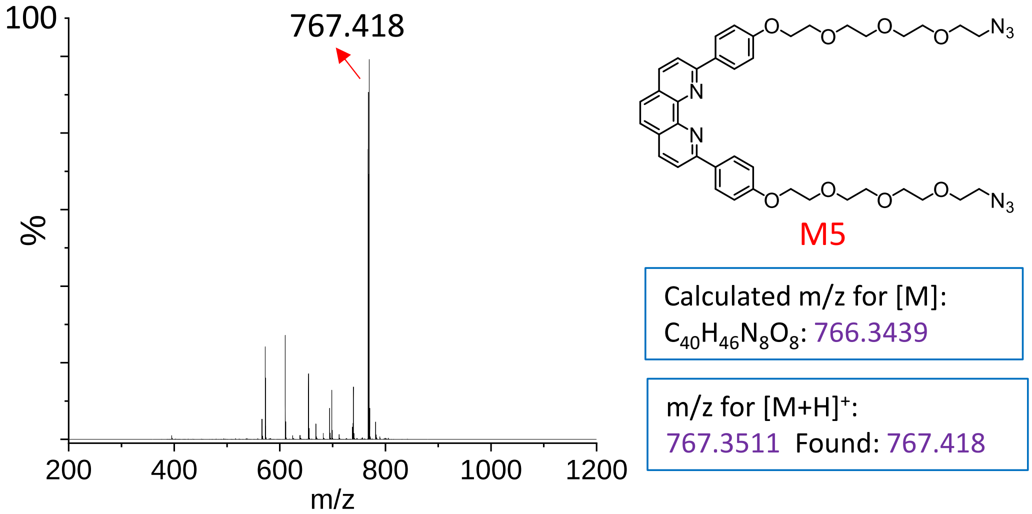

Figure S25. Mass spectrum (ESI+) analysis for di-azide monomer M5.

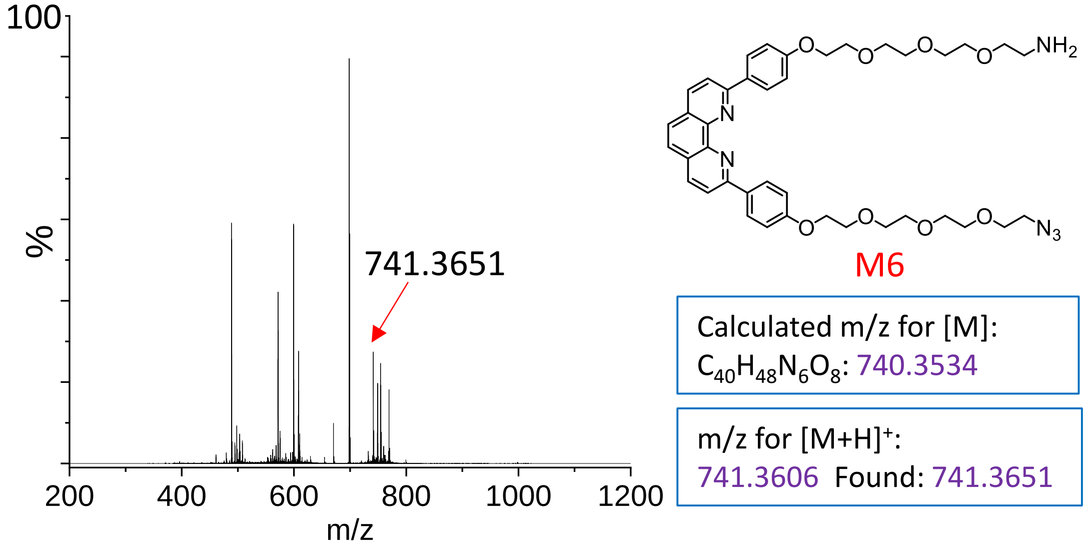

Figure S26. Mass spectrum (ESI+) analysis for mono-amine monomer M6.

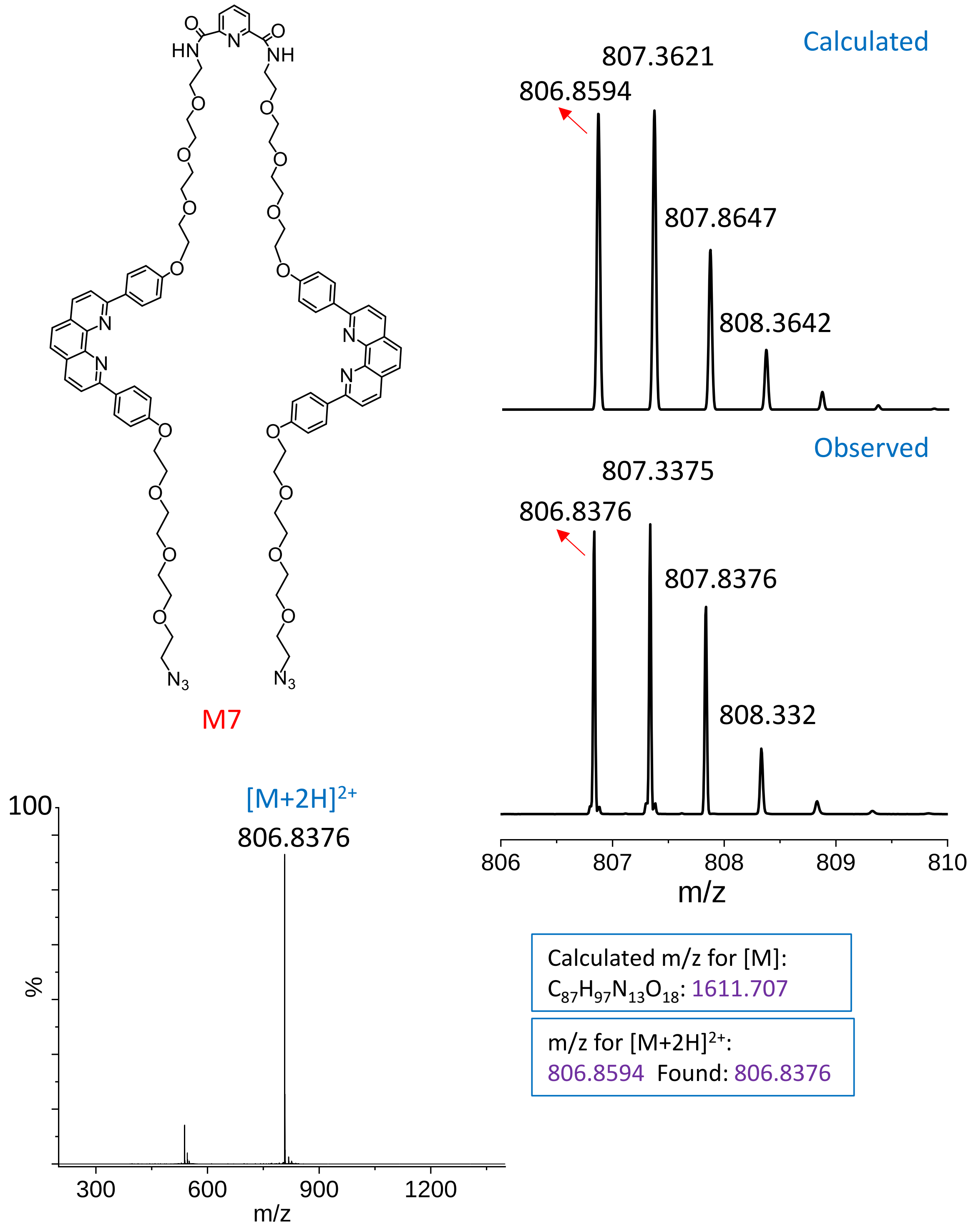

Figure S27. Mass spectrum (ESI^+) analysis for terminal di-azide monomer M7.

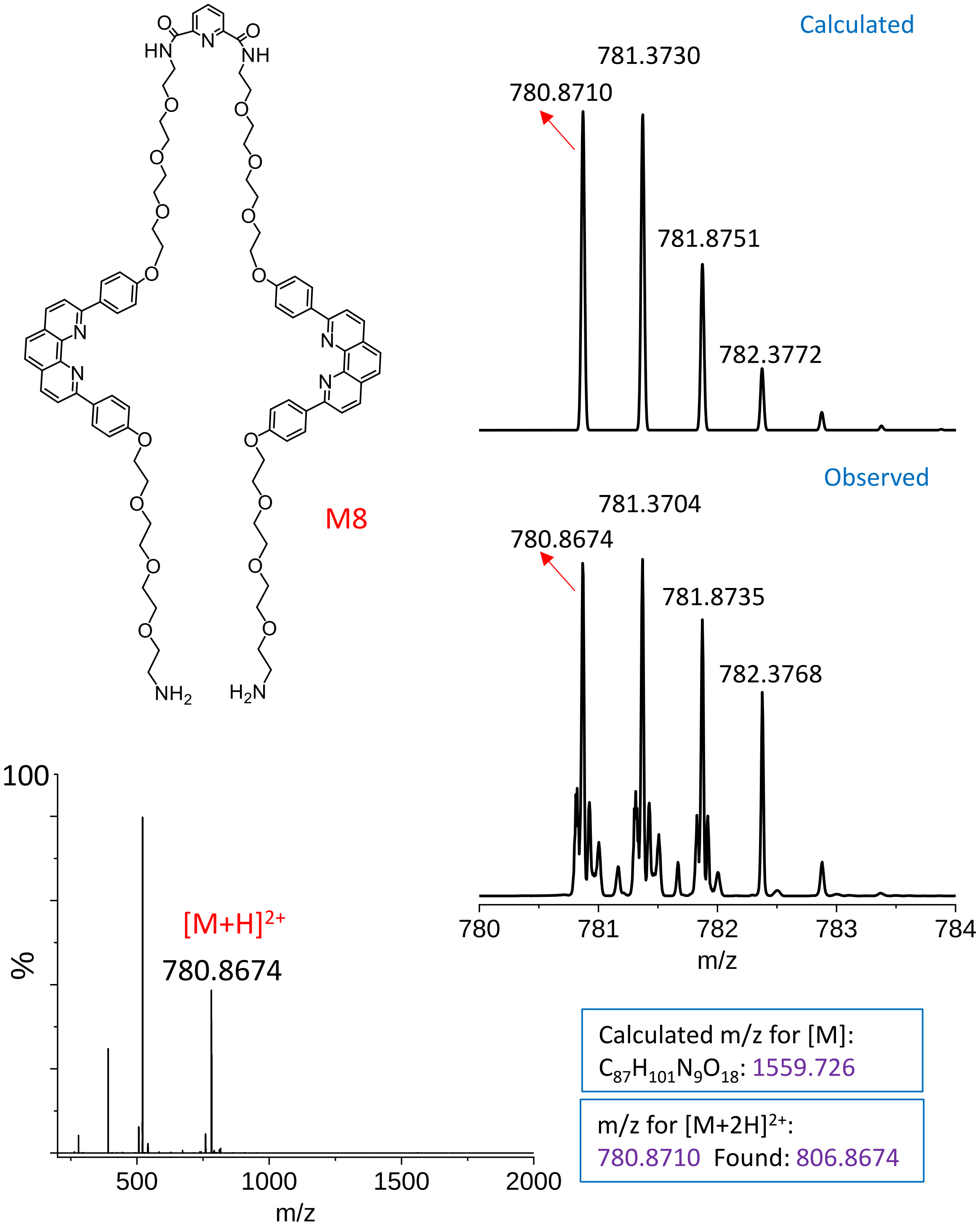

Figure S28. Mass spectrum (ESI$^+$) analysis for terminal di-amine monomer M8.

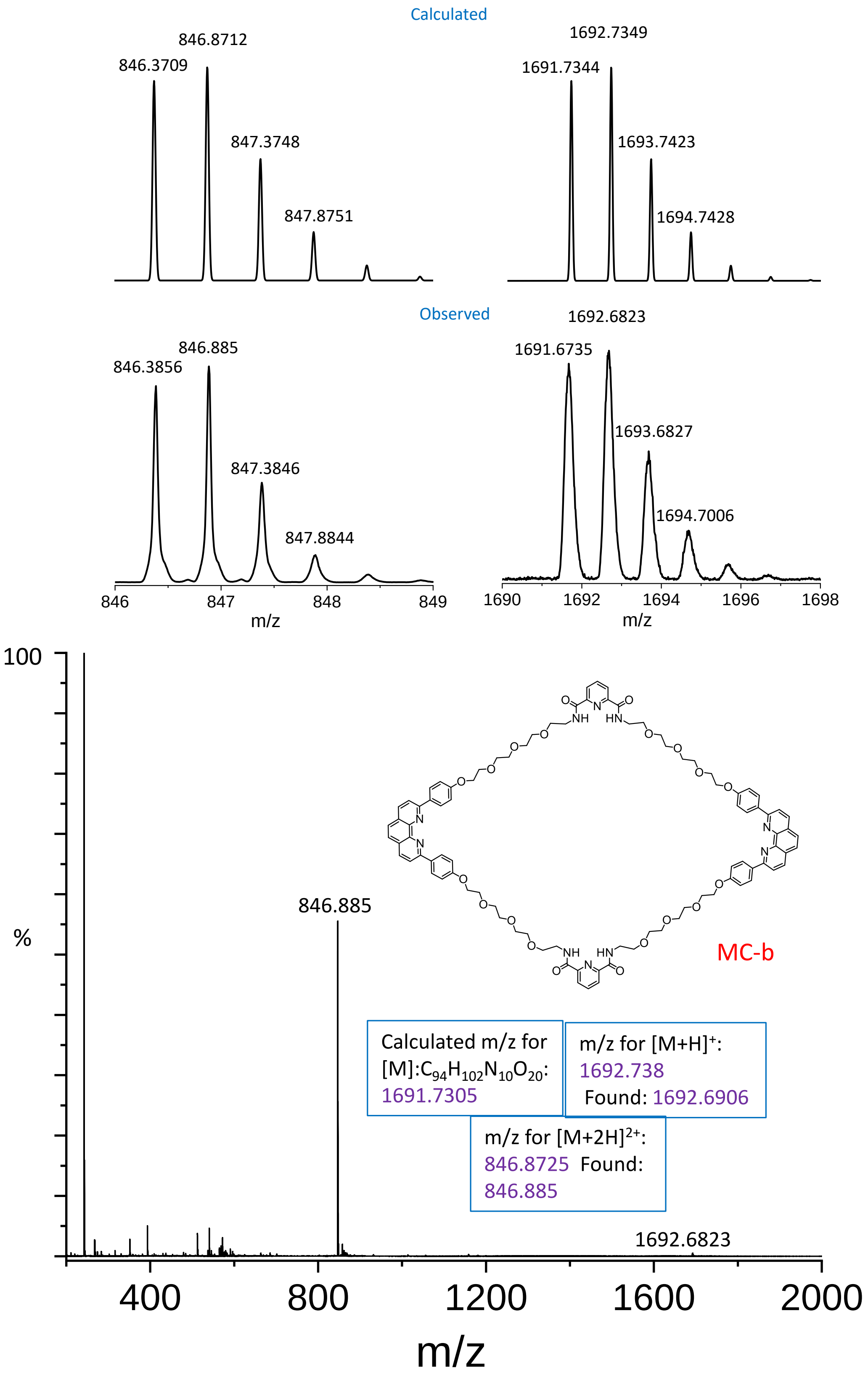

Figure S29. Mass spectrum (ESI^+) analysis for bimodal macrocycle MC-b.

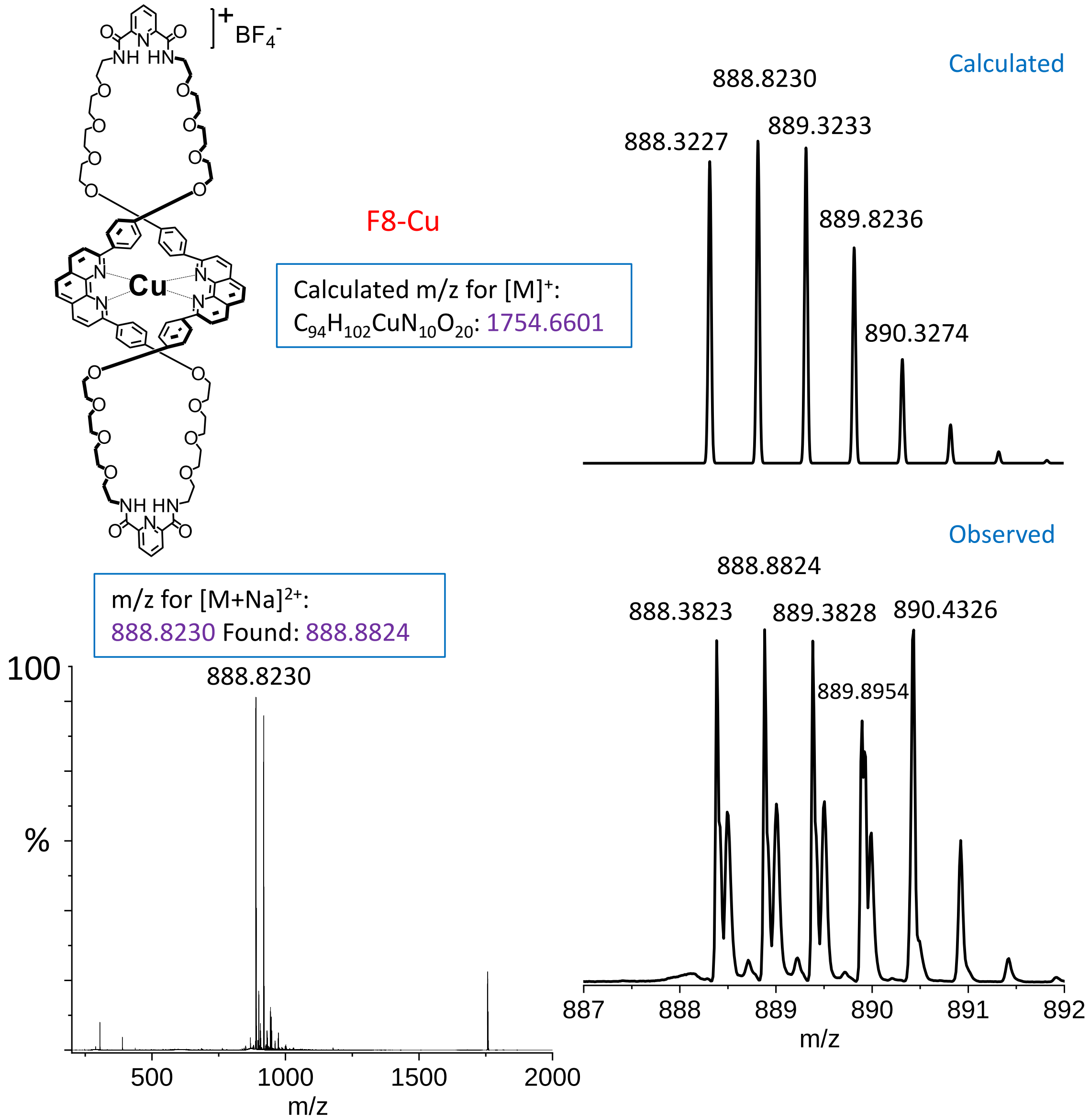

Figure S30. Mass spectrum (ESI^+) analysis for F8-Cu complex.

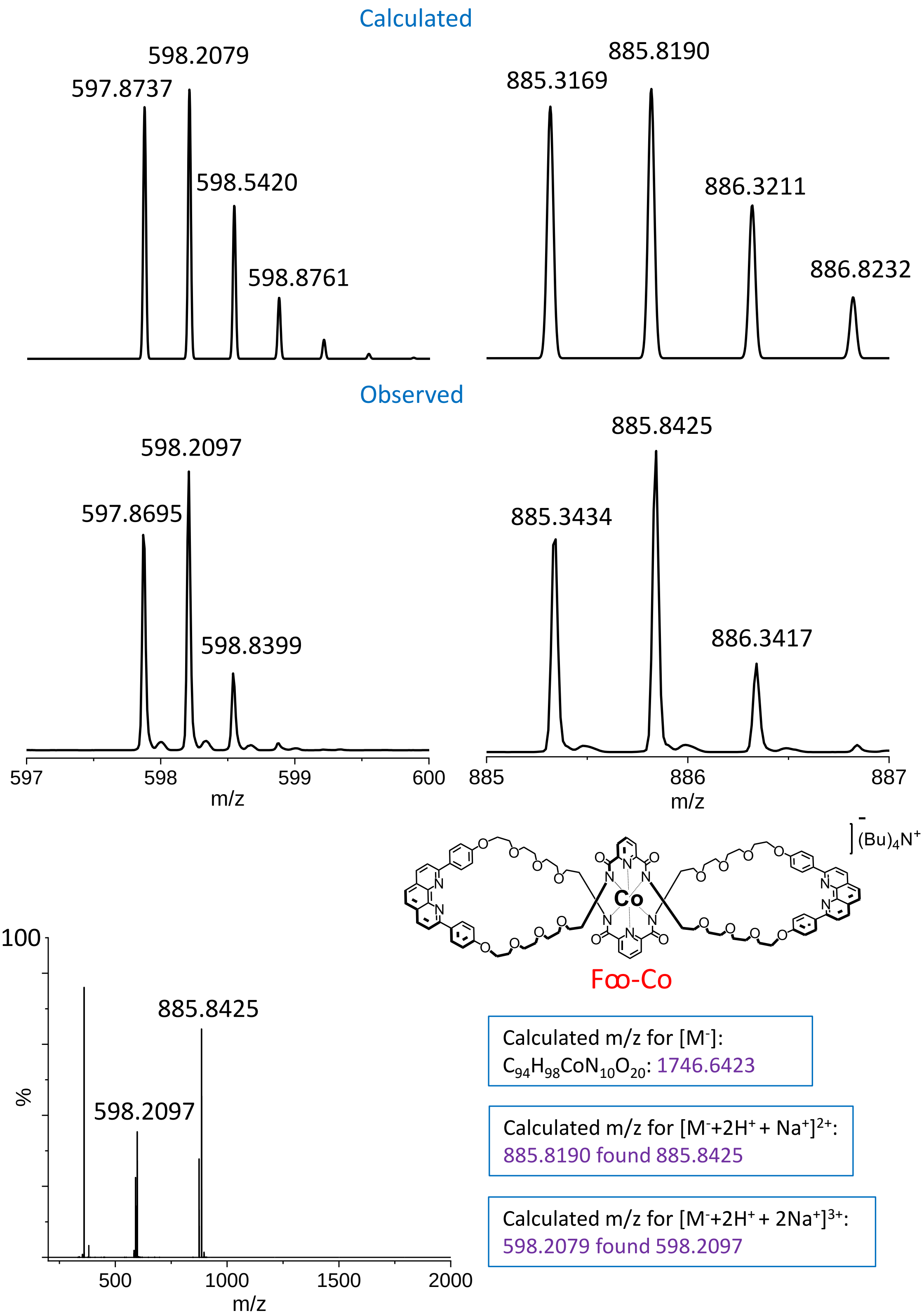

Figure S31. Mass spectrum (ESI^{+}) spectra analysis for Fꝏ-Co complex.

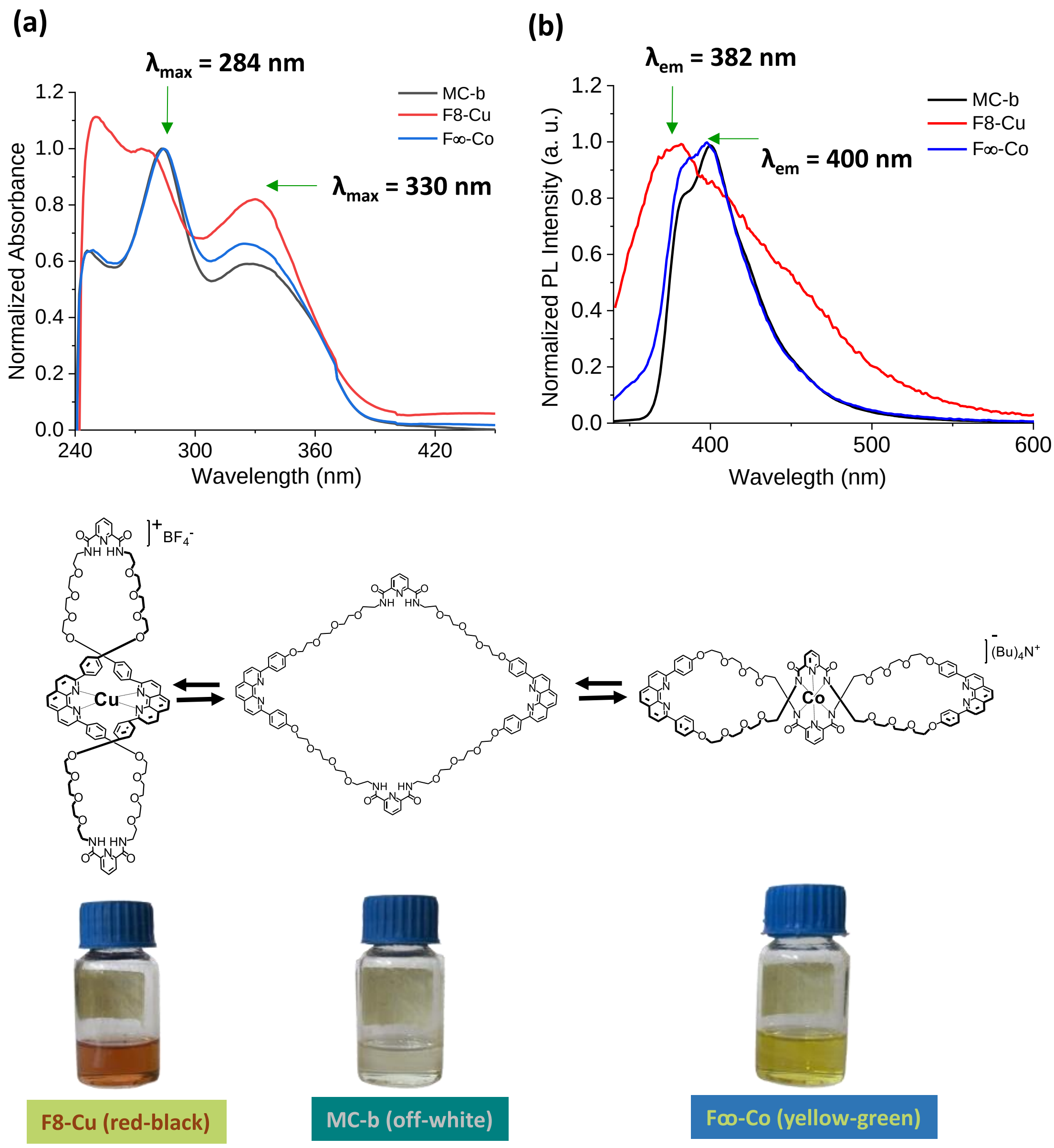

Figure S32. Optical properties of **F8-Cu**, **MC-b** and **F∞-Co** (a) UV-visible spectra (b) Fluorescence Spectra by exciting at 330 nm. Photographic images of chloroform solutions of corresponding samples are given at bottom.

References

1. C. D. Buchecker, J. P. Sauvage, *Tetrahedron*, **1990**, *46*, 503-512.
2. J. Chen, J. W. Lim, D. Y. Ong, S. Chiba, *Chem. Sci.*, **2022**, ***13***, 99-104.
3. N. D. Colley, M. A. Nosiglia, S. L. Tran, G. H. Harlan, C. Chang, R. Li, A. O. Delawder, Y. Zhang, J. C. Barnes, *ACS Cent. Sci.*, **2022**, *8*, 1672-1682.
4. K. L. Dao, R. R. Sawant, J. A. Hendricks, V. Ronga, V. P. Torchilin, R. N. Hanson, *Bioconjug. Chem.*, **2012**, *23*, 785-795.
5. M. B. Podh, R. Ratha, C. S. Purohit, *Chem. Asian. J.*, **2024**, *19*, e202400031.
6. Y. Yao, Y. Deng, L. Kong, H. Y. A. Yeung, *Eur. J. Inorg. Chem.*, **2022**, e202200271.
